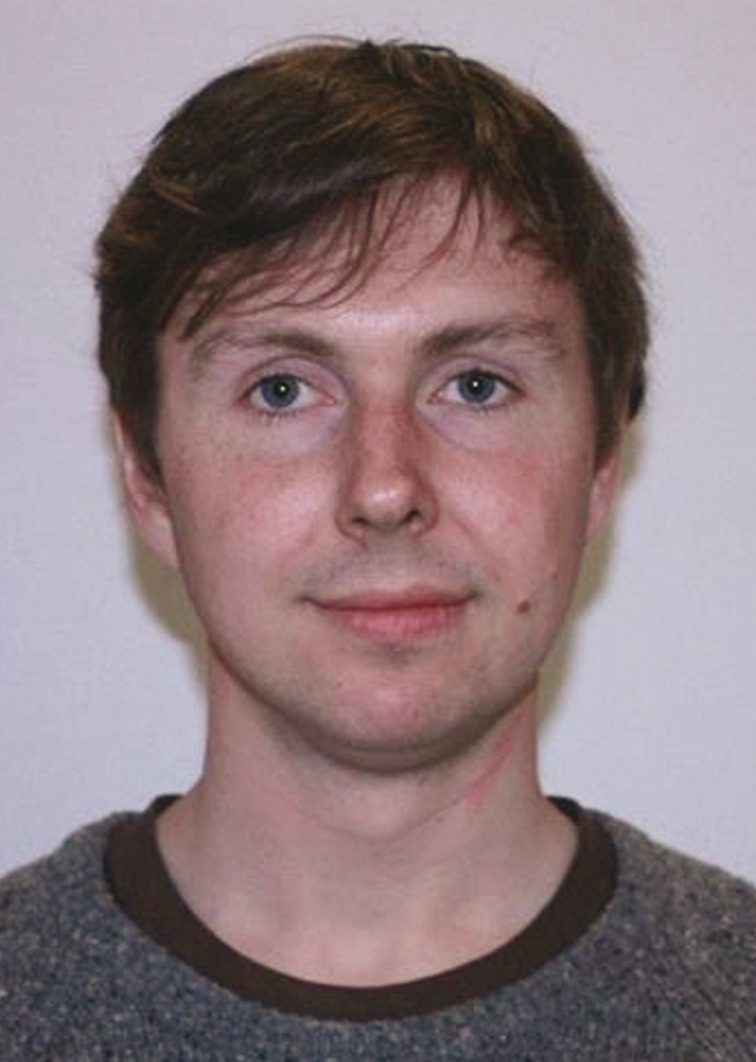# Electrophilic Aminating Agents in Total Synthesis

**DOI:** 10.1002/anie.202102864

**Published:** 2021-08-06

**Authors:** Lauren G. O'Neil, John F. Bower

**Affiliations:** ^1^ School of Chemistry University of Bristol Cantock's Close Bristol BS8 1TS UK; ^2^ Department of Chemistry University of Liverpool Crown Street Liverpool L69 7ZD UK

**Keywords:** C−N bonds, electrophilic amines, natural products, radicals, total synthesis

## Abstract

Classical amination methods involve the reaction of a nitrogen nucleophile with an electrophilic carbon center; however, in recent years, umpoled strategies have gained traction where the nitrogen source acts as an electrophile. A wide range of electrophilic aminating agents are now available, and these underpin a range of powerful C−N bond‐forming processes. In this Review, we highlight the strategic use of electrophilic aminating agents in total synthesis.

## Graphical abstract

This Review outlines the strategic use of electrophilic aminating agents in total synthesis. A wide range of C−N bond‐forming methods are highlighted, including polar, radical and transition‐metal‐catalyzed processes.

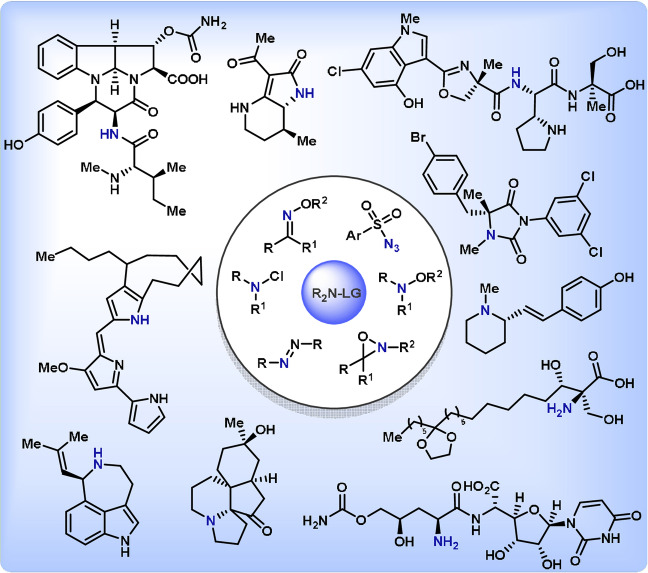

## 1 Introduction

The amine functionality is ubiquitous in nature, pharmaceuticals, agrochemicals and materials.^[1]^ The importance of nitrogen‐containing molecules is demonstrated by their large representation within FDA‐approved, small‐molecule drug compounds.^[2]^ As a result, the development of new synthetic methodologies for the construction of C−N bonds is of paramount importance.[^3^, ^4^] In recent years, significant advances in this area have been made in the fields of photoredox catalysis,[^5^, ^6^, ^7^] organocatalysis[^8^, ^9^, ^10^] and transition‐metal catalysis.[^11^, ^12^] Nevertheless, the most commonly utilized methods in total synthesis remain substitution reactions, reductive amination,[^13^, ^14^] and transition‐metal‐catalyzed C–N cross‐coupling reactions.[^15^, ^16^] These approaches all involve nucleophilic sources of nitrogen, and this can present issues in the synthesis of complex molecules. For example, the common problem of overalkylation of amine nucleophiles is often circumvented by use of protecting groups, which, in turn, means that several synthetic steps can be required to introduce one new C−N bond.

**Scheme 1:**
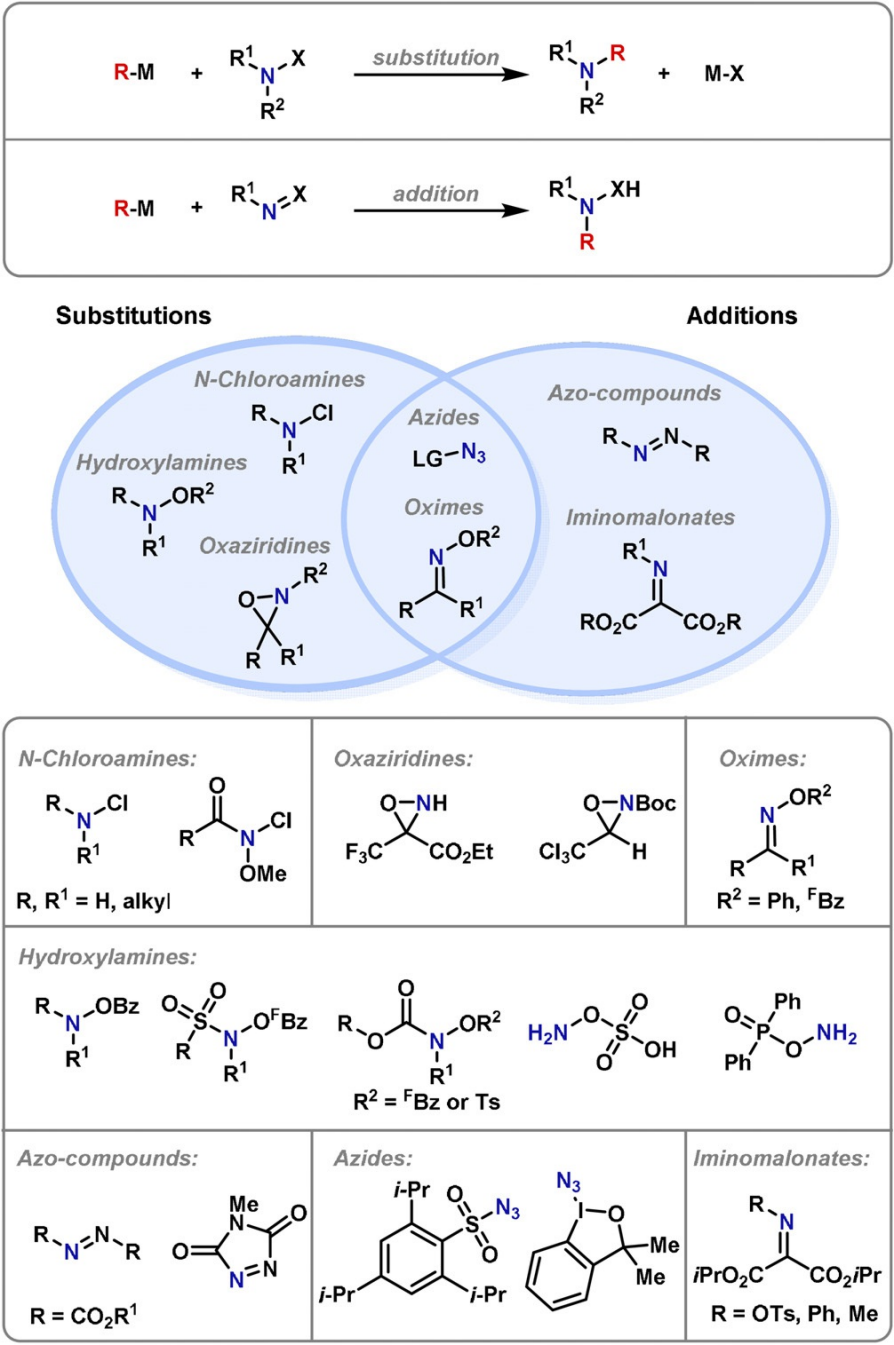
Electrophilic aminating agents. Boc=*tert*‐butyloxycarbonyl, Bz=benzoyl, ^F^Bz=pentafluorobenzoyl, LG=leaving group, Ts=toluenesulfonyl.

More recently, efforts have been directed towards the development of umpoled strategies that employ electrophilic sources of nitrogen (i.e. equivalents to the R_2_N^+^ synthon). This approach addresses problems associated with nucleophilic nitrogen‐based strategies and has several advantages, notably the ability to functionalize typically unreactive bonds. A large proportion of electrophilic amination reactions are carried out with a preformed electrophilic aminating agent, and due to the topical interest in this strategy, a plethora of variants are now readily available (Scheme 1). Electrophilic aminating agents can be split into two distinct classes: those that undergo substitution reactions, and those that undergo addition reactions. Certain reagents can participate in both mechanistic regimes. For substitution reactions, the general structure of the aminating reagent is R_2_N‐X, where X is an electron‐withdrawing group, which is displaced by nucleophilic attack or bond cleavage. The most commonly used N‐based electrophiles for substitutions are N‐chloroamines,[^17^, ^18^] oxaziridines[^19^, ^20^] and hydroxylamines.[^21^, ^22^] Examples of electrophilic aminating agents that tend to undergo addition processes include azo compounds[^23^, ^24^] and iminomalonates,^[25]^ whilst azides[^26^, ^27^] and oximes^[28]^ are able to engage in both.

Until the last decade, the use of electrophilic aminating agents had received little attention; however, there are now several excellent reviews outlining the multitude of transformations that can be achieved.[^29^, ^30^, ^31^, ^32^, ^33^, ^34^, ^35^] This review aims to give an overview of the burgeoning field of electrophilic amination by presenting the most relevant examples of total syntheses involving *preformed* electrophilic aminating agents. For the purposes of this review, electrophilic amination with nitrene species will not be discussed; the reader is instead directed to several comprehensive reviews for further information on this topic.[^36^, ^37^, ^38^, ^39^, ^40^, ^41^, ^42^, ^43^]

## 2 α‐Amination of Carbonyl Compounds

**Scheme 2:**
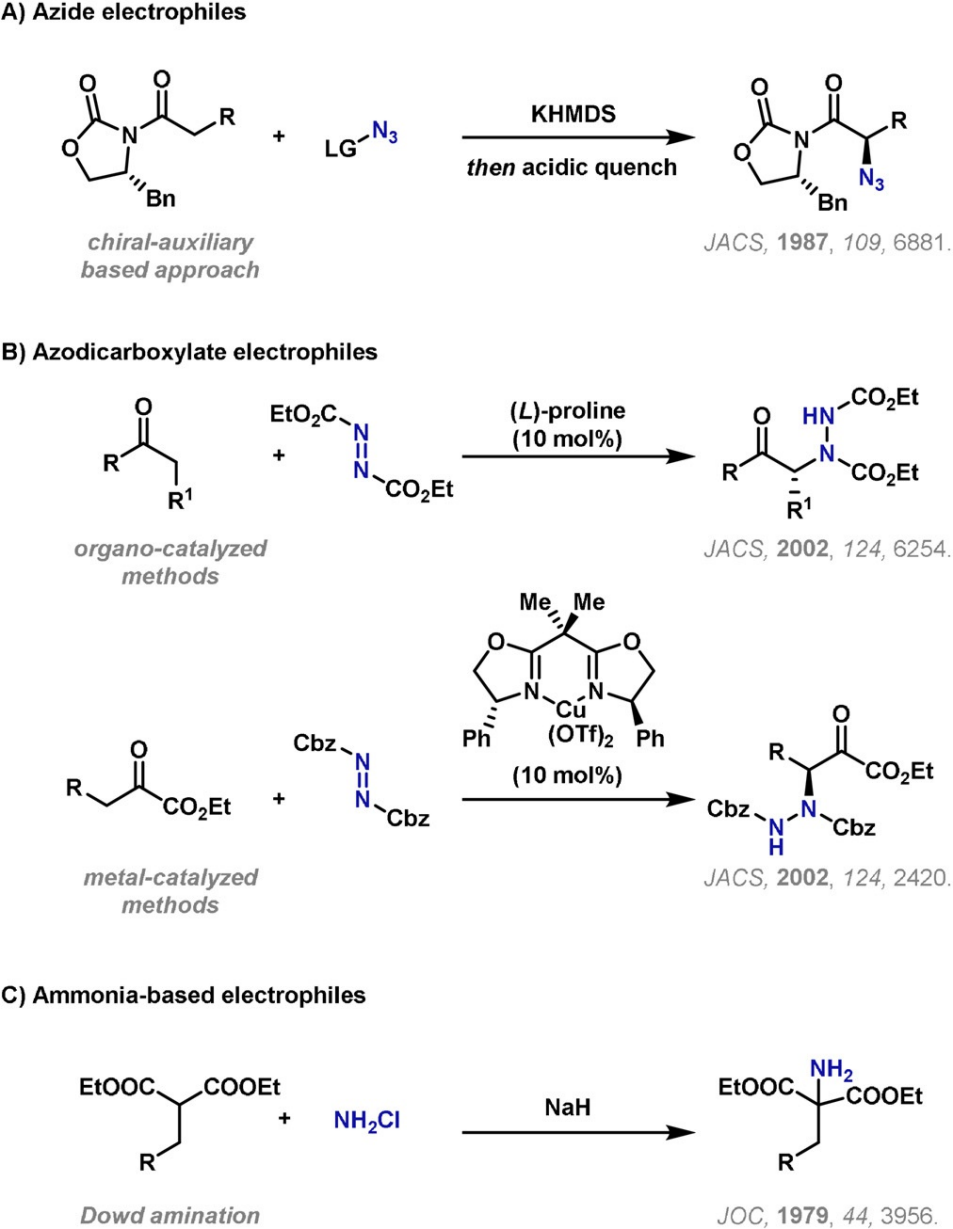
Electrophilic amination methods for the α‐functionalization of carbonyl compounds. Bn=benzyl, Cbz=benzyloxycarbonyl, KHMDS=potassium bis(trimethylsilyl)amide, Tf=trifluoromethanesulfonyl.

The development of methods for electrophilic amination adjacent to a carbonyl group has been of significant interest as it allows the synthesis of α‐amino acid derivatives, a common feature of many bioactive molecules. The asymmetric synthesis of quaternary amino acids is relatively challenging and is commonly carried out by chiral‐auxiliary‐controlled Strecker syntheses, or by diastereoselective alkylation of chiral enolates.[^44^, ^45^, ^46^, ^47^, ^48^, ^49^, ^50^] However, these methods usually require a protecting group strategy when applied to the synthesis of a complex molecule. The use of electrophilic aminating agents can avoid this, and such transformations can be carried out asymmetrically, using either a chiral auxiliary or chiral catalyst (Scheme 2). The formation of a C−N bond in this manner constitutes one of the simplest methods to establish a stereogenic carbon center of this type, and a number of total syntheses have incorporated this methodology as a key step.

The α‐amination of carbonyl compounds can be achieved either via a preformed enolate or via direct α‐amination using an organocatalyst or metal catalyst.[^17^, ^26^, ^51^, ^52^, ^53^, ^54^, ^55^, ^56^, ^57^] A wide range of electrophilic aminating agents have been used in these processes to generate a plethora of products with new C−N bonds (Scheme 2). For this type of transformation, an amine is rarely installed directly; however, there are several reported methods that allow the one‐ or two‐step conversion of the initial adduct into either the corresponding amine or another synthetically useful N‐based functionality. The most commonly utilized electrophiles in total syntheses are azides and azo compounds. These reagents are often commercially available, and the resulting azide or hydrazine products are readily manipulated.

### 2.1 Azide Electrophiles

A large range of azide electrophiles are commercially available or readily prepared.^[58]^ α‐Amination using these reagents produces a new azide (Scheme 2 A), which can serve as a versatile handle for further manipulation. For example, the azide group can be readily converted to the corresponding amine by reduction.^[59]^ The electrophilic azidation process usually requires the preformation of an enolate nucleophile, and the aminating agent is typically equipped with an electron‐withdrawing sulfonyl unit, which activates it and is removed at the end of the reaction. Asymmetric versions of this process typically employ a chiral auxiliary on the enolate partner.^[26]^ Although the use of a chiral auxiliary is suboptimal, the versatility of the resulting azide group and the ability to construct a functionalized stereocenter offers significant benefits. Additionally, this approach can provide complementary access to diazo compounds via judicious choice of enolate precursor, electrophilic azide source and workup procedure.^[60]^

**Scheme 3:**
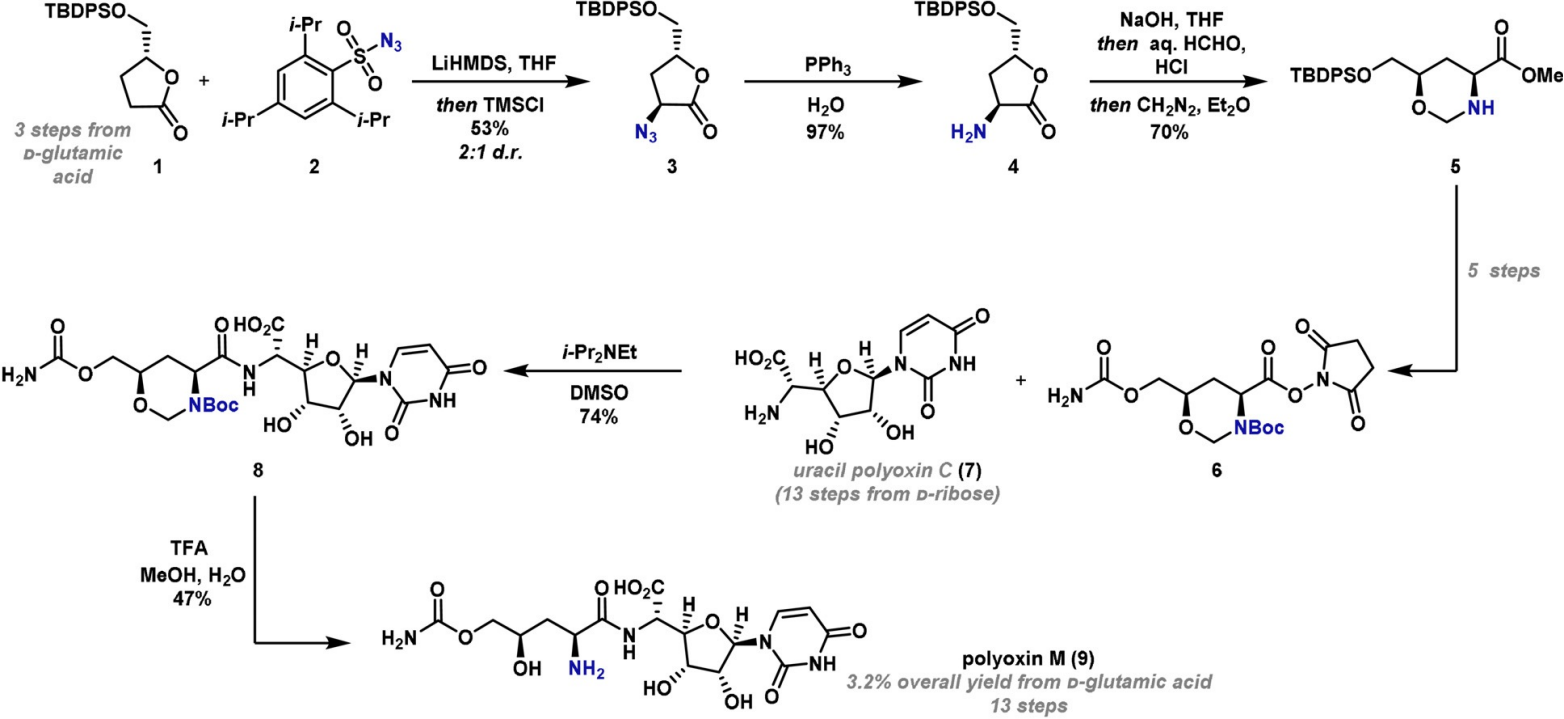
Akita's synthesis of polyoxin M (**9**). DMSO=dimethyl sulfoxide, d.r.=diastereomeric ratio, LiHMDS=lithium bis(trimethylsilyl)amide, TBDPS=*tert*‐butyldiphenylsilyl, THF=tetrahydrofuran, TFA=trifluoroacetic acid, TMS=trimethylsilyl.

In 2006, Akita reported the first synthesis of the peptidyl nucleoside antibiotic polyoxin M (**9**), where a substrate‐controlled diastereoselective electrophilic azidation was employed as a key step (Scheme 3).^[61]^ Substituted lactone **1** was synthesized in three steps from commercially available d‐glutamic acid. It was postulated that the bulky TBDPS protecting group of **1** could be used to direct the azidation of the enolate from the opposite face. An electrophilic azidation reaction was chosen in preference to other electrophilic C−N bond formations because earlier work on a similar substrate found that high diastereoselectivities could be achieved with this approach.[^26^, ^62^] In the event, treatment of the lithium enolate of **1** with trisyl azide **2** provided azide product **3** in 53 % yield, but with modest diastereoselectivity (2:1 *d.r*.). Subsequent Staudinger reduction of azide **3** gave free amine **4** in excellent yield. This was advanced to **5** via a three‐step sequence involving hydrolysis of the lactone, acid‐mediated formation of the 1,3‐oxazinane (HCl, HCHO) and methyl esterification. A further five steps gave active ester **6**, which was coupled via amide bond formation with uracil polyoxin C (**7**), previously synthesized in thirteen steps from d‐ribose.[^63^, ^64^] Treatment of the resulting amide **8** with TFA effected removal of the N‐Boc and N,O‐acetal protecting groups to give polyoxin M (**9**) in 47 % yield. Although the electrophilic azidation step (**1** → **3**) suffered from poor diastereoselectivity, it did enable the establishment of a key stereocenter in a direct and simple manner, and it provides a notable example of the use of a substrate‐controlled azidation reaction in total synthesis.

**Scheme 4:**
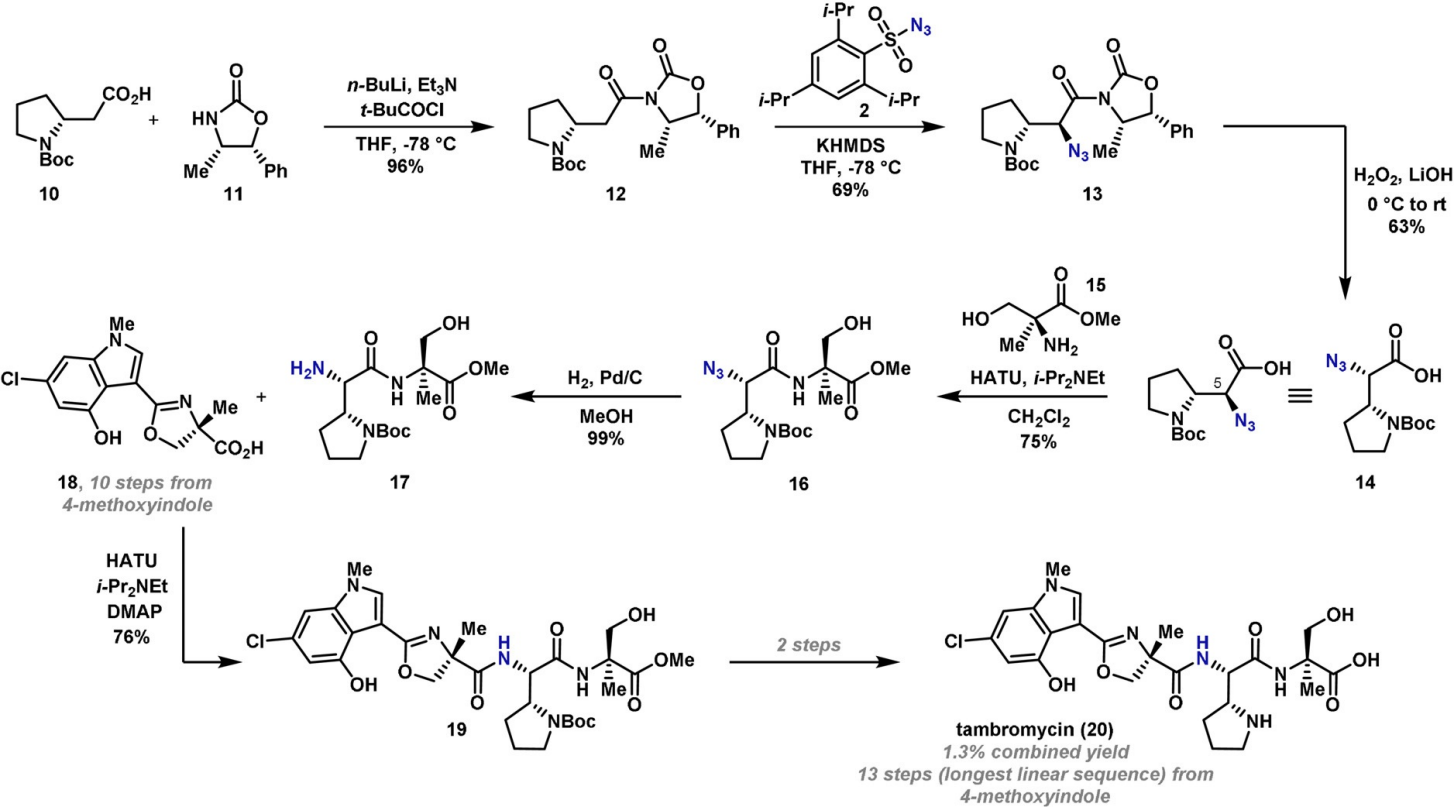
Thomson's synthesis of tambromycin (**20**). DMAP=4‐dimethylaminopyridine, HATU=1‐[bis(dimethylamino)methylene]‐1*H*‐1,2,3‐triazolo[4,5‐b]pyridinium 3‐oxide hexafluorophosphate, rt=room temperature.

The diastereoselectivity of the azidation reaction in Akita's synthesis is under substrate control; however, auxiliary‐controlled processes have also been exploited. In 2018, Thomson reported the synthesis of the tetrapeptide tambromycin (**20**), where one of the peptide units was installed by a chiral‐auxiliary‐controlled diastereoselective electrophilic azidation reaction (Scheme 4).^[65]^

Retrosynthetically, it was postulated that fragments **17** and **18** could be combined by amide coupling, and therefore efforts centered on their synthesis. The indole fragment **18** was synthesized in ten steps from commercially available 4‐methoxyindole. The synthesis of fragment **17** began with the installation of the chiral auxiliary, which was necessary to achieve good diastereoselectivity in the electrophilic azidation reaction. Acylation of **10** with oxazolidinone **11** gave **12** in excellent yield. After extensive optimization, it was found that reaction of **12** with KHMDS and electrophilic aminating agent **2** gave the desired azide product **13** in good yield and, importantly, as a single stereoisomer. Removal of the chiral auxiliary with lithium hydrogen peroxide gave acid **14** in good yield, and epimerization of the potentially labile C(5) stereocenter was not observed. Amide coupling of **14** with α‐l‐methyl‐serine methyl ester **15** gave amide **16**, and the azide was subsequently reduced by hydrogenation to give **17** in excellent yield. Following coupling of **17** and **18**, **19** was advanced to tambromycin (**20**) in two further steps, with the synthesis completed in thirteen steps (longest linear sequence) and a combined yield of 1.3 %.

Although auxiliary‐based, the conversion of **12** to **13** by electrophilic azidation provided a single diastereomer, which would have been challenging to achieve by other methods. From a strategic viewpoint, it is also important to recognize that conventional methods for accessing peptides often require the installation and removal of a protecting group on nitrogen. An electrophilic azidation strategy can help minimize protecting group manipulations. Recently, catalyst‐controlled electrophilic α‐azidation protocols have been developed, and these remove the need for a chiral auxiliary. For example, it has been shown that chiral iron or copper catalysts can promote highly enantioselective electrophilic azide transfers.[^27^, ^66^] In these processes, the electrophilic aminating agent is an azide‐containing hypervalent iodine(III) species. These species are often highly unstable; however, it has been shown that safer variants can be developed.^[67]^ At the current stage, these catalyst‐controlled α‐azidation processes are relatively substrate specific, although their development bodes well for future applications in total synthesis. Advances in this area will be important for enhancing electrophilic amination as a synthetic design strategy and maintaining its competitiveness versus other contemporary approaches. For example, in the case of tambromycin (**20**), Renata subsequently disclosed a competitive synthetic route that exploits advances in biocatalytic C–H functionalization.^[68]^ The use of enzymes in this case, removed the need for a chiral auxiliary during the assembly of the right‐hand fragment, **17**.

### 2.2 Azodicarboxylate Electrophiles

**Scheme 5:**
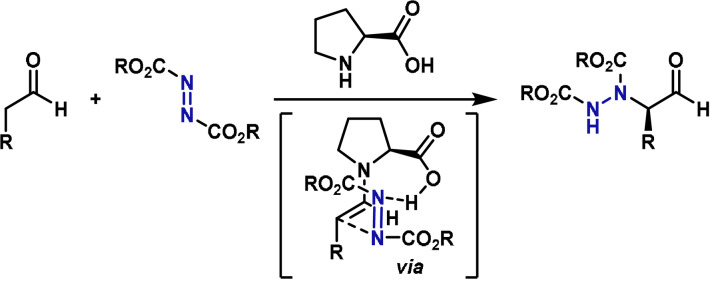
Proposed transition state for proline‐catalyzed α‐amination with azodicarboxylate electrophiles.

In the context of total synthesis, azodicarboxylate aminating agents are by far the most popular electrophiles for the α‐amination of carbonyl compounds.[^69^, ^70^, ^71^, ^72^, ^73^, ^74^, ^75^, ^76^, ^77^] This is because they are commercially available, relatively bench stable, and highly electrophilic. Chiral organocatalysts and chiral auxiliary‐based methods have been developed that provide high enantioselectivities. For the former, the high efficiency of proline‐based catalysts can be rationalized by the transition state indicated in Scheme 5; here, approach of the azodicarboxylate is directed by hydrogen bonding between one of its nitrogen centers and the carboxylic acid unit of the organocatalyst. This model is based on Houk and List's calculated transition state of the Hajos–Parrish–Eder–Sauer–Wiechert reaction, and is analogous to transition states that have been proposed for proline‐catalyzed intermolecular Mannich and aldol reactions.[^23^, ^78^]

There are several complementary methods for the cleavage of the N−N bond of the initial hydrazine product. Classical reduction conditions, such as Raney nickel,^[79]^ SmI_2_,^[80]^ and Na/NH_3_,^[81]^ are most commonly used and have been applied in several total syntheses. More recently, a milder (non‐reductive) method has been reported, wherein the N−N bond is cleaved via an E1cB process.^[82]^ The development of other mild N–N cleavage methods would be beneficial for applications of azodicarboxylate‐based aminations in total synthesis.

**Scheme 6:**
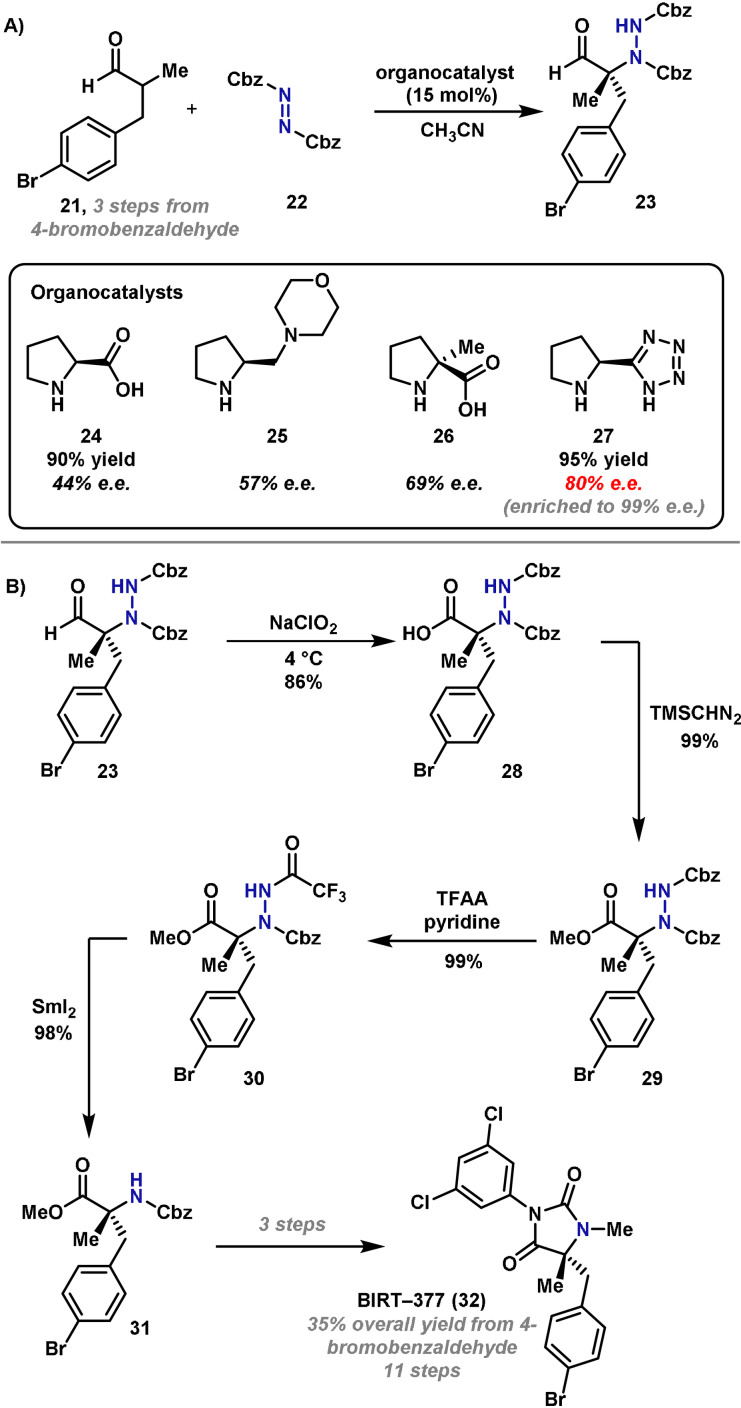
Barbas’ synthesis of cell adhesion inhibitor BIRT‐377 (**32**). *ee*=enantiomeric excess, TFAA=trifluoroacetic anhydride.

In 2004, Barbas reported the total synthesis of the cell adhesion inhibitor BIRT‐377 (**32**), where the key tetrasubstituted stereocenter was introduced by an asymmetric organocatalyzed electrophilic α‐amination (Scheme 6).^[83]^ The electrophilic amination required the enantioselective reaction of aldehyde **21** (prepared in three steps) and a dibenzyl azodicarboxylate electrophile, **22**; to achieve this, a range of chiral organocatalysts were investigated (Scheme 6 A). The use of l‐proline (**24**)^[84]^ offered only modest enantioinduction (44 % *ee*) and required a prolonged reaction time of 5 days. Moderate improvements were observed with (*S*)‐4‐(pyrrolidin‐2‐ylmethyl)morpholine (**25**) (with TFA as an additive), which provided **23** in 57 % *ee*, whereas α‐methyl‐l‐proline **26** offered a further increase to 69 % *ee*. Finally, by changing to tetrazole catalyst **27**,^[85]^ an 80 % *ee* was achieved, and this could be improved to >99 % *ee* following recrystallization. The high enantioselectivity observed with tetrazole catalyst **27** in comparison to that with proline **24** was attributed to the difference in p*K*_a_ and increased steric bulk. Tetrazole catalyst and l‐proline have p*K*_a_ values of 8 and 12 in DMSO, respectively,^[86]^ and therefore, the hydrogen‐bonding interactions involving **27** and **24** are different, resulting in different enantioselectivities.

With **23** in hand, a seven‐step sequence was developed to complete the synthesis. Oxidation of amino aldehyde **23** gave the corresponding carboxylic acid **28** (Scheme 6 B), which was treated with (trimethylsilyl)diazomethane to give ester **29**. Cleavage of the N−N bond of **29** proved challenging, as treatment under reductive conditions with SmI_2_ was unsuccessful. It is known that trifluoroacetylated hydrazines undergo N−N bond cleavage with SmI_2_;^[87]^ therefore **29** was converted to trifluoroacetylated hydrazine **30**. Selective N−N bond cleavage with SmI_2_ was then achieved to give quaternary amino acid derivative **31**. Cbz deprotection, hydantoin formation and N‐methylation gave BIRT‐377 (**32**), with an overall yield of 35 % from 4‐bromobenzaldehyde.

The Barbas synthesis of BIRT‐377 (**32**) showcases the use of organocatalyzed α‐amination methodology for the introduction of a new tetrasubstituted stereocenter in a complex molecule. Notably, although the key C−N bond‐forming process is efficient, the cleavage of the N−N bond of **29** was challenging and required several steps. Consequently, methods that can address this inefficiency would be desirable. Alternate syntheses of BIRT‐377 (**32**) have employed either phase‐transfer catalysis or a chiral pool approach involving Seebach's self‐regeneration of chirality as a means of introducing the key stereocenter.[^88^, ^89^, ^90^] Barbas’ electrophilic aminating‐based approach compares favorably to these other syntheses and also allows the easy preparation of analogues.

**Scheme 7:**
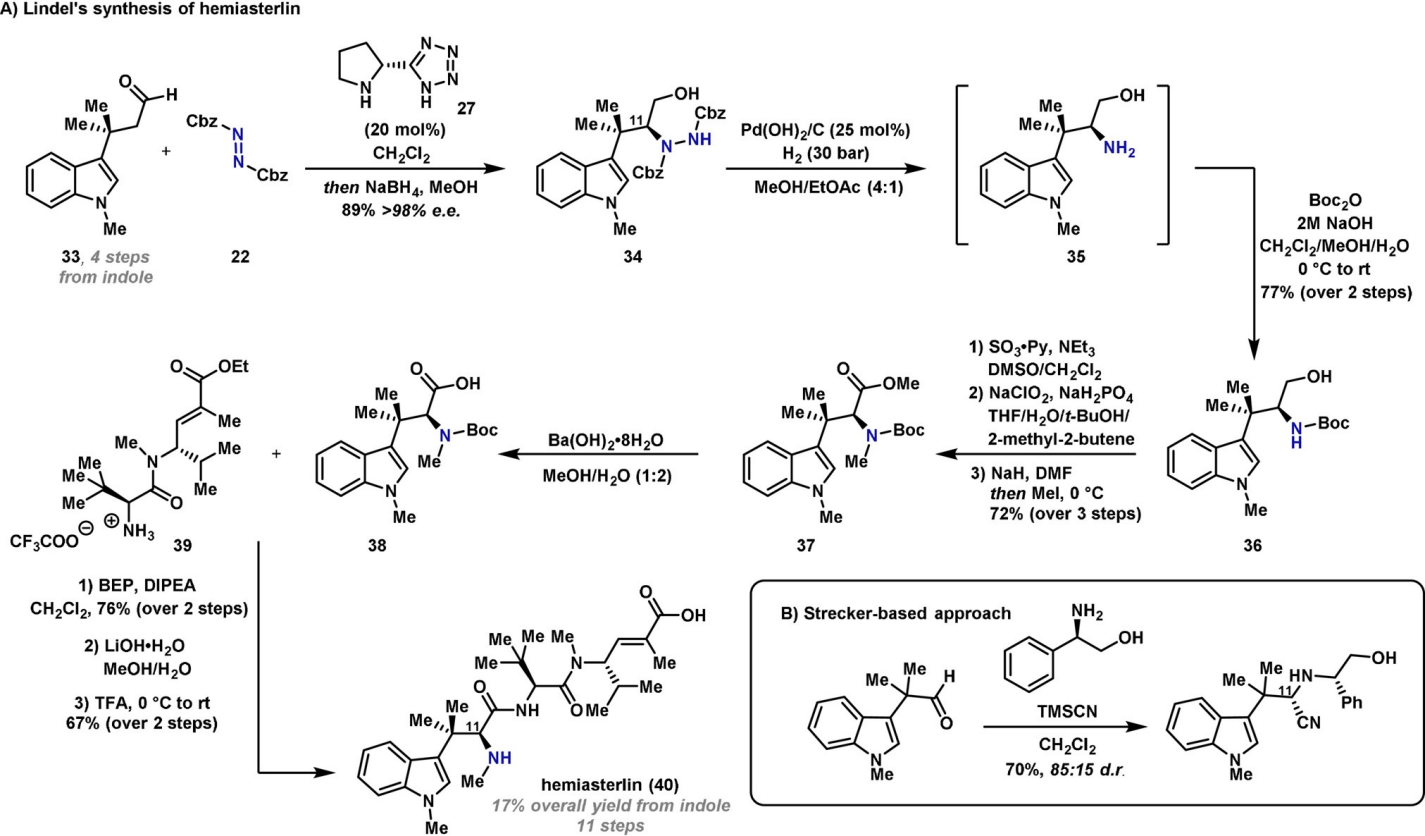
Lindel's synthesis of marine natural product, hemiasterlin (**40**). BEP=2‐bromo‐1‐ethylpyridinium tetrafluoroborate, DIPEA=*N*,*N*‐diisopropylethylamine, DMF=*N*,*N*‐dimethylformamide, Py=pyridine.

The combination of the dibenzyl azodicarboxylate electrophile **22** and tetrazole organocatalyst **27** can also be used to access tertiary amino acids. In 2017, Lindel and co‐workers exploited this for the introduction of a key stereocenter during an enantioselective synthesis of the marine natural product hemiasterlin (**40**) (Scheme 7 A).^[91]^ Aldehyde **33** was synthesized in four steps from indole, where the side chain was introduced by a Pd‐catalyzed C(3)‐selective allylation reaction.^[92]^ α‐Amination using azodicarboxylate **22** and organocatalyst **27** proceeded in excellent yield, and after aldehyde reduction, alcohol **34** was isolated in 98 % *ee*. Initial investigations for the cleavage of the N−N bond of **34** focussed on the aforementioned E1cB method; however, **35** was not observed and so hydrogenolytic methods were explored. Hydrogenation of alcohol **34** with Raney Ni, Pd/C or PtO_2_ was unsuccessful. N–N cleavage was observed when polymethylhydrosiloxane (PMHS) was employed as a “green source” of hydrogen in combination with a PdCl_2_ catalyst;^[93]^ however, this approach was unreliable. The use of Pearlman's catalyst (Pd(OH)_2_/C) in EtOAc/MeOH (4:1) at 30 bar was found to be optimal and gave amine **35**, which was immediately protected to afford **36** in excellent yield. This was advanced in a further three steps to deliver tryptophan **37**. Hydrolysis of the methyl ester to give **38** was followed by the known amide coupling with dipeptide **39**.^[94]^ Finally, ester hydrolysis and N‐Boc deprotection gave hemiasterlin (**40**) in an overall yield of 17 %.

The Lindel synthesis of hemiasterlin (**40**) demonstrates the advantages of using electrophilic α‐amination methodology as a means of synthesizing chiral α‐amino acid derivatives. Although there have been several previously reported syntheses of hemiasterlin, a significant disadvantage of these approaches is that they involve a chiral auxiliary, thereby mandating additional auxiliary installation and removal steps.[^94^, ^95^, ^96^, ^97^] In most of these cases, the introduction of the amino‐bearing C(11) stereocenter was achieved by auxiliary‐controlled asymmetric Strecker reaction, as summarized in Scheme 7 B.[^95^, ^97^]

**Scheme 8:**
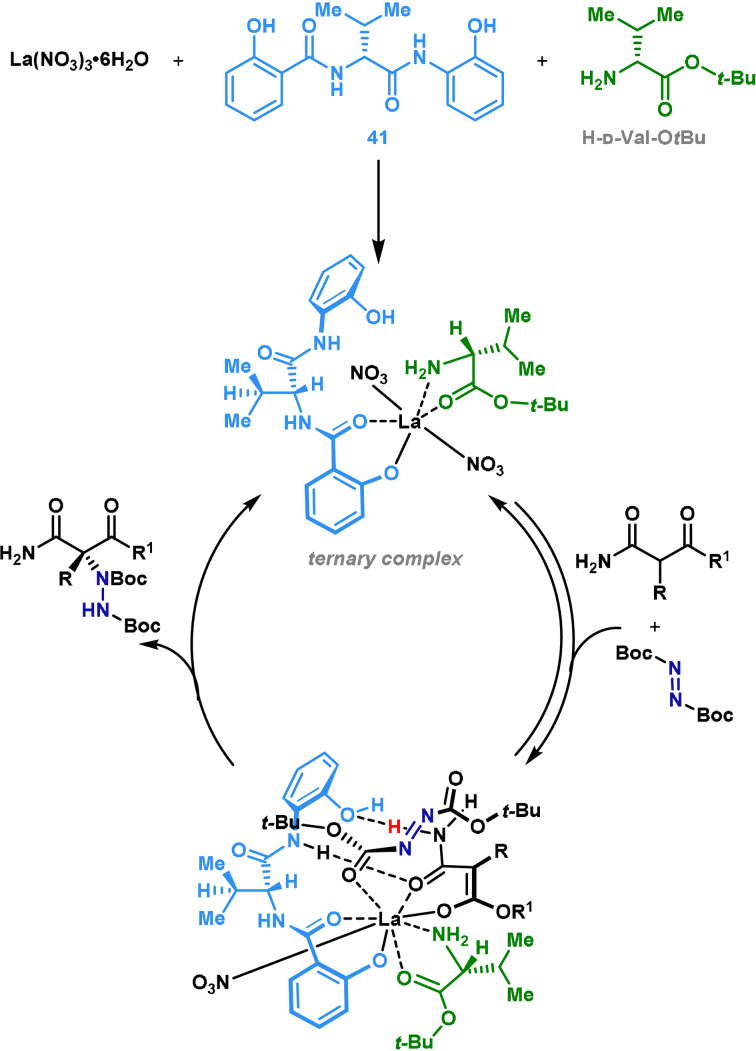
Proposed catalytic cycle for Shibasaki's asymmetric α‐amination.

Most examples of azodicarboxylate‐based enantioselective α‐aminations of carbonyl compounds use a chiral organocatalyst, but complementary transformations can be achieved with a transition‐metal catalyst.[^57^, ^98^, ^99^, ^100^, ^101^] This allows the installation of C−N bonds in more sterically demanding systems, as well as enabling direct C‐H amination processes. Shibasaki and co‐workers have reported a procedure for the α‐amination of carbonyl compounds using a catalyst system comprising La(NO_3_)_3_⋅6 H_2_O, an amide‐based ligand (**41**) and d‐valine‐*tert*‐butyl ester.[^100^, ^102^, ^103^] It is postulated that the three catalyst components are in dynamic equilibrium and work in a synergistic manner (Scheme 8). This method is particularly suited to enantioselective aminations of nonprotected substrates containing (mild) Lewis basic functionality, for example, succinimide derivatives and secondary α‐alkoxycarbonyl amides. Subunits of this type are common building blocks in total synthesis and feature in many natural products.[^104^, ^105^] In similar processes where an organocatalytic approach is employed, substrates with multiple coordination sites can result in poor enantioselectivity. Therefore, this methodology constitutes a powerful option for the construction of densely functionalized α‐amino acid derivatives.

**Scheme 9:**
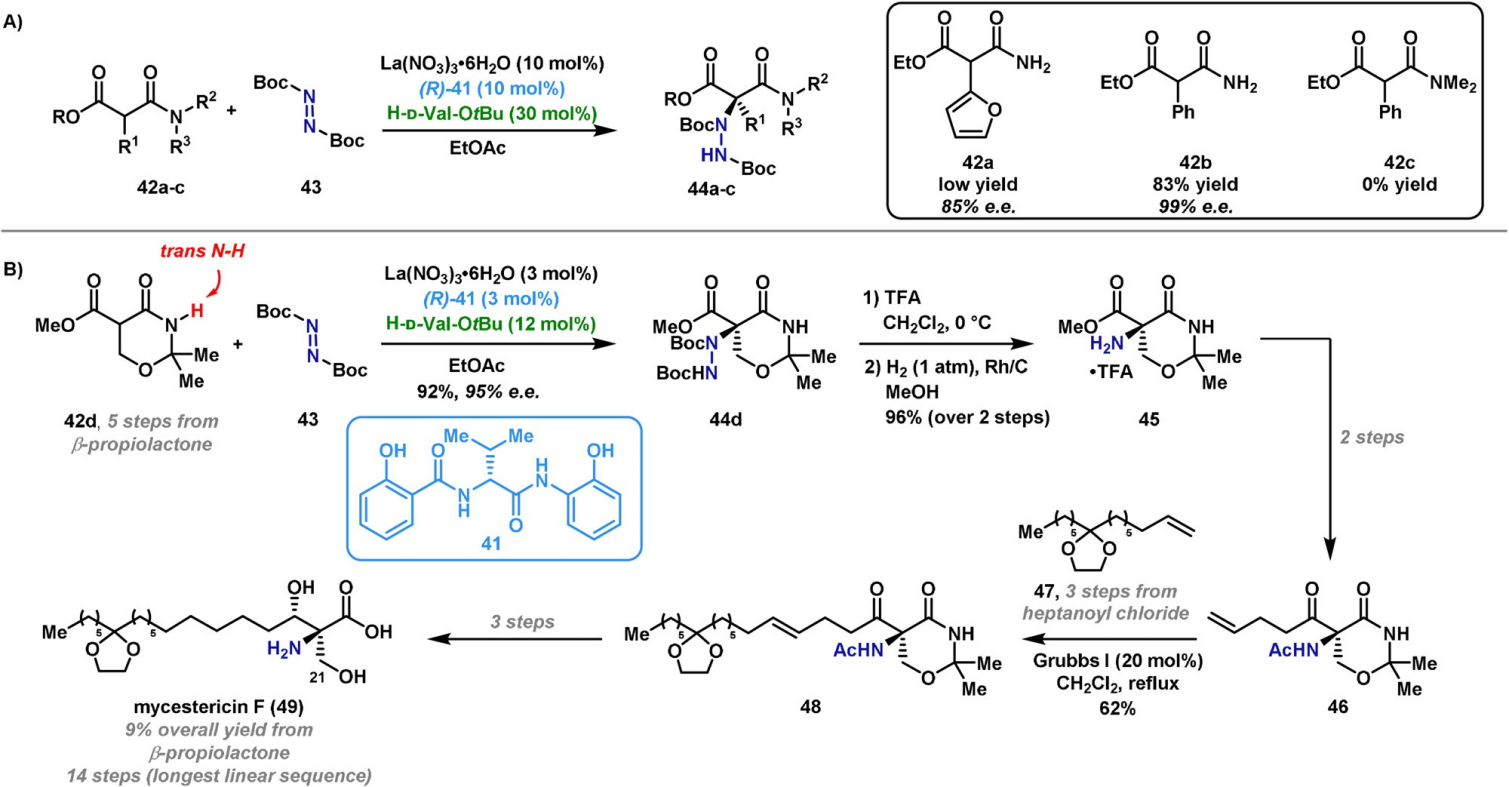
Shibasaki's synthesis of mycestericin F (**49**).

In 2011 Shibasaki and co‐workers reported the application of their methodology to the synthesis of mycestericin F (**49**),^[103]^ which is a potent immunosuppressant (Scheme 9).^[106]^ It was postulated that the “polar head” of the molecule could be installed by an asymmetric electrophilic α‐amination of an amide using the ternary catalytic system shown in Scheme 8. Other syntheses of this unit require lengthy synthetic routes as a result of the challenges associated with the highly functionalized stereocenter.[^107^, ^108^, ^109^] Shibasaki's studies began with the evaluation of several substrates (selected examples are shown in Scheme 9 A) for the key electrophilic amination; the substrates examined all possessed an sp^2^‐based R^1^ substituent that could undergo subsequent oxidation to introduce the C(21) hydroxyl group of the target. Upon treatment with the ternary catalytic system and di‐*tert*‐butyl azodicarboxylate electrophile **43**, it was found that only substrate **42 b** gave the desired amination product in acceptable yield and enantioselectivity. It was postulated that a *trans* N–H proton is required on the α‐alkoxycarbonyl amide motif to obtain high enantioselectivity, as this facilitates favorable hydrogen‐bonding interactions with the catalyst, as shown in Scheme 8.^[100]^ Substrate **42 d** was later designed as a viable intermediate for the synthesis of mycestericin F (**49**) (Scheme 9 B). The optimized conditions for the key α‐amination require use of H‐d‐Val‐O*t*Bu to ensure a high level of stereocontrol, and control experiments confirmed that every component of the catalyst system is required. Other known organo‐ and metal‐based catalytic systems were also investigated;[^54^, ^110^, ^111^, ^112^] however, comparable levels of enantioinduction were not observed in most cases. With **44 d** in hand, a sequence of N‐Boc deprotection (TFA) and hydrogenolytic N–N bond cleavage provided amine **45**. Conventional hydrogenation catalysts, such as Raney Ni and Pd/C, were unsuccessful; it was eventually found that N–N cleavage could be achieved in good yield by use of Rh/C under an atmospheric pressure of hydrogen. Amine **45** was then advanced over two steps to metathesis precursor **46**. The aliphatic tail, **47**, was synthesized in three steps from commercially available *n*‐heptanoyl chloride, following previously reported procedures.^[113]^ Cross‐metathesis of **46** and **47** (1:3 molar ratio) was executed by exposure to the Grubbs first generation catalyst, which provided **48** in 62 % yield. A further three steps were required to access mycestericin F (**49**) in good overall yield.

**Scheme 10:**
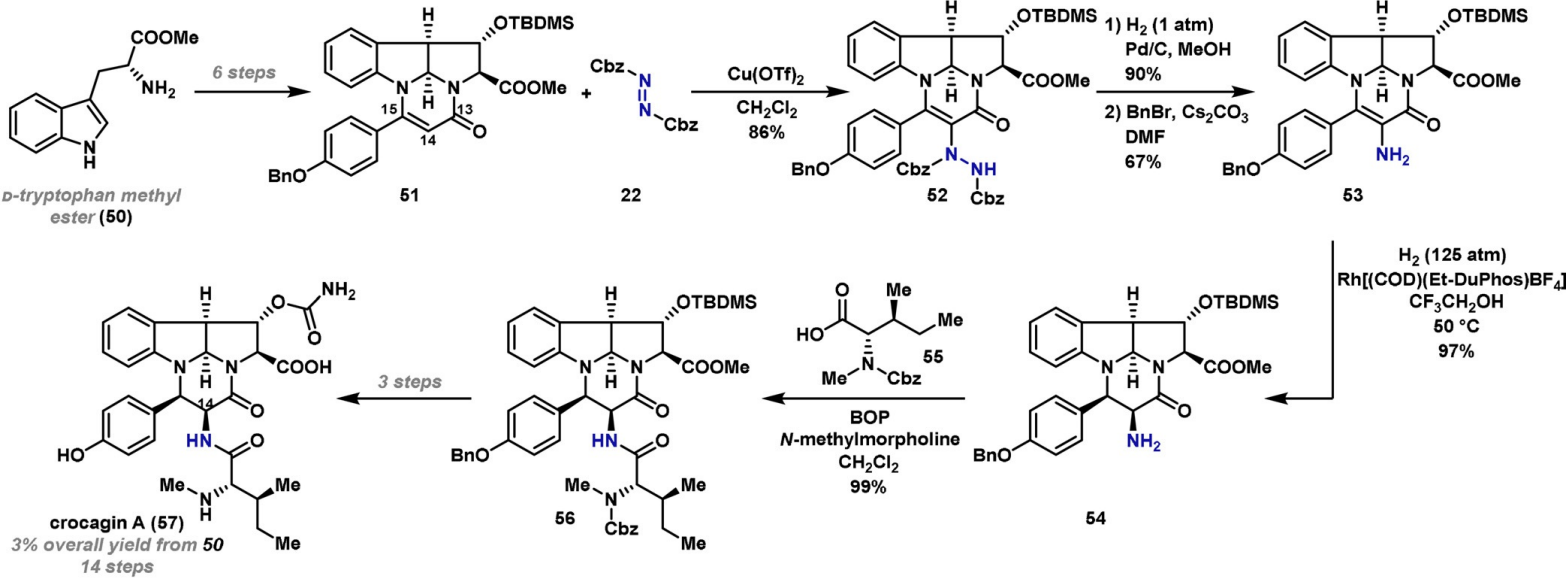
Trauner's synthesis of crocagin A (**57**). BOP=benzotriazol‐1‐yloxytris(dimethylamino)phosphonium hexafluorophosphate, Rh[(COD)(Et‐DuPhos)BF_4_]=1,2‐bis[2,5‐diethylphospholano]benzene(1,5‐cyclooctadiene)rhodium(I) tetrafluoroborate, TBDMS=*tert*‐butyldimethylsilyl.

The Shibasaki method involves catalyst‐promoted enolization. The direct electrophilic amination of other types of enolate equivalents can also be achieved under metal‐catalyzed conditions; for example, in certain contexts, enol ethers and related systems can be aminated using silver‐ or copper‐based catalysts.[^99^, ^101^] Trauner and co‐workers have demonstrated the power of this approach in the synthesis of crocagin A (**57**), a bioactive compound isolated from myxobacterium *Chondromyces crocatus* (Scheme 10).^[114]^

It was hypothesized that the C(14)−N bond of **57** could be installed by electrophilic amination, prior to introduction of the *N*‐methyl isoleucine side chain, **55**. To execute this strategy, pyrimidinone **51** was identified as a suitable precursor, and this was prepared in six steps from **50**. The installation of the desired C−N bond to **51** was challenging, likely due to the diminished nucleophilicity of the C(14)–C(15) alkene double bond as a result of conjugation with the C(13) carbonyl. Indeed, several azodicarboxylate electrophiles were investigated without success. Subsequently, it was found that amination could be achieved using copper(II) triflate as a Lewis acidic catalyst in combination with dibenzyl azodicarboxylate **22**, providing **52** in 86 % yield. N–N cleavage and global benzyl deprotection under hydrogenation conditions gave amine **53** in good yield after reinstallation of the benzyl ether. From here, the C(14)–C(15) alkene double bond was reduced under substrate control[^115^, ^116^] to give amine **54** with excellent diastereoselectivity. The *N*‐methyl isoleucine unit **55** was then installed by amide coupling to give peptide **56**, which was advanced in a further three steps to crocagin A (**57**). This synthesis showcases the power of functionalizing readily assembled unsaturated ring systems prior to strategic (stereocenter‐installing) reduction processes.

### 2.3 Electrophilic Ammonia‐Based Reagents

Ammonia‐based electrophiles are an alternative class of aminating agent that have received some attention in recent years as the NH_2_ functionality can be directly installed. This offers significant advantages compared to azodicarboxylate and azide electrophiles, which require post C−N bond‐formation manipulations to reveal the NH_2_ moiety. A commonly exploited electrophile for ammonia‐based processes is monochloramine (NH_2_Cl), which can be prepared as a solution in Et_2_O by reaction of NH_4_Cl, NH_4_OH and bleach.[^117^, ^118^] Mechanistically, electrophilic α‐aminations with NH_2_Cl are thought to proceed via direct nucleophilic attack of an enolate onto the electrophilic nitrogen center.

**Scheme 11:**
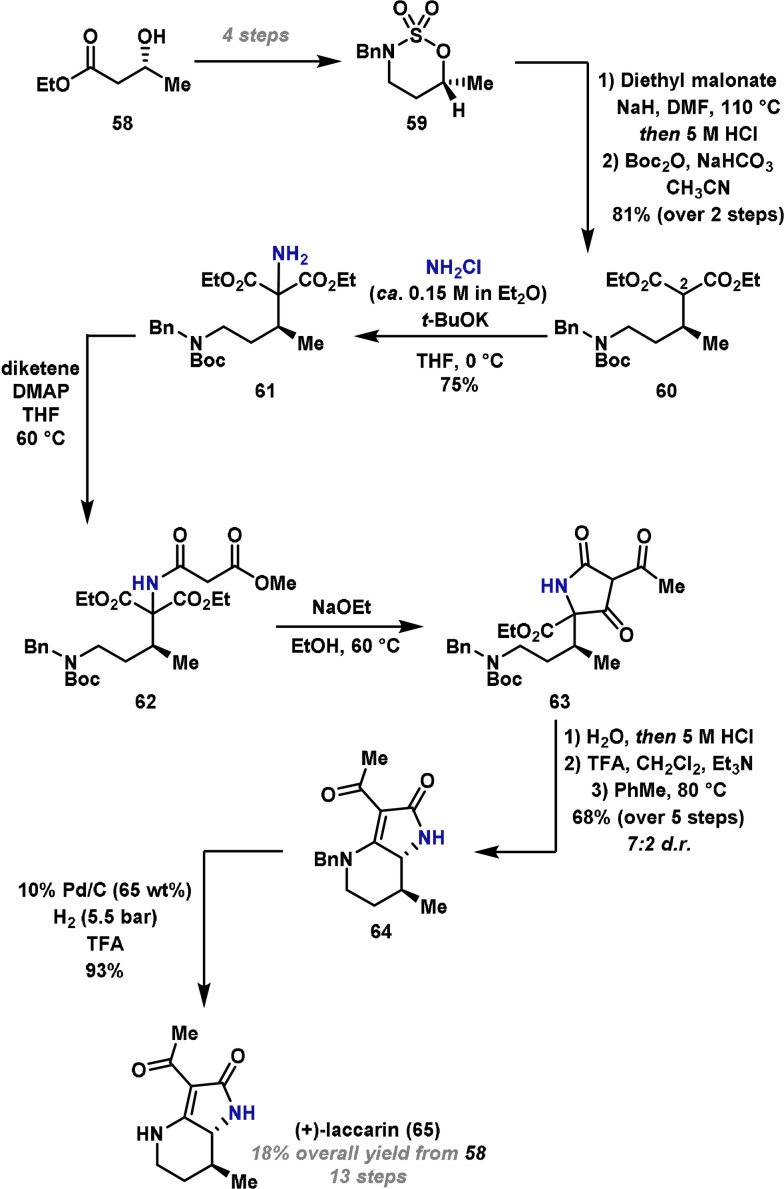
Gallagher's synthesis of (+)‐laccarin (**65**).

In 2004, Gallagher and co‐workers reported a short and efficient enantioselective synthesis of (+)‐laccarin (**65**), a fungal metabolite with a densely functionalized piperidine core.^[119]^ It was proposed that the five‐membered ring could be accessed by a three‐step approach involving an electrophilic amination with NH_2_Cl to introduce the required amine, followed by a tandem acylation and cyclization sequence. Studies commenced with preparation of cyclic sulfamidate **59** in four steps from commercially available ethyl (*3R*)‐hydroxybutyrate **58** (Scheme 11). Sulfamidate **59** was treated with the sodium enolate of diethyl malonate, which resulted in stereoinvertive ring cleavage at the O‐bearing carbon. In situ acid‐promoted cleavage of the N‐sulfate leaving group and Boc‐protection of the benzylamine unit gave **60** in 81 % yield (over two steps). The latter step was required to prevent lactamization. The installation of the C−N bond at C(2) was then achieved in good yield by exposure of the potassium enolate of **60** to ethereal NH_2_Cl. Acylation of the resulting amine **61** with diketene was followed by cyclization of **62** under Claisen conditions to provide amide **63**. Saponification of the ester, acid‐promoted decarboxylation and N‐Boc deprotection allowed cyclocondensation to afford **64** in 7:2 *d.r*. The synthesis was completed by hydrogenolytic removal of the N‐benzyl group, which afforded (+)‐laccarin (**65**) in 93 % yield.

The Gallagher laccarin synthesis utilizes the Dowd amination reaction,^[17]^ which exploits one of the cheapest and most simple electrophilic aminating agents, NH_2_Cl. Examples of the large‐scale use of this reagent are relatively scarce;^[117]^ this likely reflects its instability and toxicity. However, the ability to directly install an NH_2_ unit is notable and contrasts other electrophilic α‐amination reactions discussed so far. In the case of (+)‐laccarin, the diastereoselectivity associated with the new C−N bond is likely under thermodynamic control. To advance the utility of this approach, it would be desirable to develop catalyst‐controlled α‐amination reactions that use safe and stable alternatives to monochloramine. For example, methoxyamine (NH_2_OMe) is an alternative reagent that enables the installation of NH_2_ units in other contexts. Under basic conditions, this reagent promotes the stereoretentive conversion of pinacol boronate esters to primary amines.[^120^, ^121^] Here, the electrophilicity associated with the N−O bond facilitates C−N bond formation. Jin, Liu and co‐workers have developed a related method that exploits the reactivity of H_2_N‐DABCO, an aminoazanium aminating agent.^[122]^ One could envisage developing protocols that exploit these reagents for the α‐amination of carbonyl compounds.

The examples outlined in this section highlight the versatility of electrophilic aminating agents in the α‐amination of carbonyl compounds. Going forward, several drawbacks must be addressed to enhance the utility of this approach in synthesis. The aminating agents used for these processes are often toxic and/or unstable, and these considerations necessitate handling precautions. Additionally, many of the examples described use azodicarboxylate or azide electrophiles, which require subsequent steps to access the target amine. Nonetheless, electrophilic aminations of this type offer one of the most powerful methods for the de novo construction of amino acid units in complex molecules.

## 3 Aziridination of Olefins

Aziridines are versatile synthetic intermediates and are also found in natural products and bioactive molecules;[^123^, ^124^, ^125^] consequently, continuing efforts are aimed at developing methods for their synthesis. Aziridines provide an attractive strategy for installing amines in target‐directed synthesis because their opening with nucleophiles usually occurs in a stereocontrolled manner.[^126^, ^127^, ^128^, ^129^, ^130^, ^131^, ^132^] The synthesis of aziridines from olefins is particularly powerful because olefinic starting materials are often commercially available or simple to synthesize.[^133^, ^134^] In certain cases, the direct synthesis of amines from alkenes can be achieved in one pot via the intermediacy of an aziridine. Such processes are complementary to the α‐aminations already outlined, as they allow access to alternate substitution patterns and enable the stereoselective installation of contiguous functionality.

**Scheme 12:**
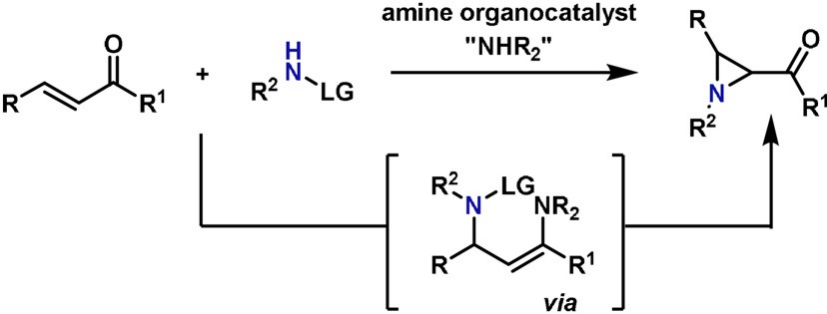
Aziridination by an aza‐MIRC type process.

The preparation of aziridines from olefins using electrophilic aminating agents is now firmly established, and as a result, methods of this type are well documented in total synthesis.^[37]^ Most of these processes involve nitrene intermediates, which, as already mentioned, are not discussed in this review. Alternatively, electrophilic aziridinations can be carried out by aza‐Michael initiated ring closure (aza‐MIRC) processes involving electron‐poor alkenes.[^135^, ^136^, ^137^, ^138^] Organocatalyzed variants of this reaction use an amine catalyst to activate an enone[^139^, ^140^] or an enal[^135^, ^141^, ^142^] via the corresponding iminium ion. This leads to an enamine intermediate which undergoes ring closure onto the electrophilic nitrogen center to provide the product (Scheme 12). Thus, aminating agents used in these processes are ambiphilic, functioning as a nucleophile for the first C−N bond formation and as an electrophile for the second. Chiral organocatalysts can be used to achieve enantioinduction.^[143]^ The most common aminating agents are hydroxylamine derivatives, in particular, N‐tosyloxycarbamates, some of which are commercially available. Accordingly, most aza‐MIRC aziridinations provide carbamate‐protected aziridines, which is beneficial as this type of protecting group can be selected for easy removal.

In 2011, Hamada reported the formal synthesis of (−)‐agelastatin A (**73**),^[144]^ a tetracyclic alkaloid with potent antiproliferative activity towards human cancer cells.[^145^, ^146^] The key feature of the molecule is the structurally complex, tetracyclic core ring system, which possesses four contiguous nitrogen‐substituted stereocenters. There have been several previous syntheses of (−)‐agelastatin A (**73**); most of these used appropriate chiral starting materials.[^147^, ^148^, ^149^, ^150^, ^151^, ^152^, ^153^, ^154^] Consequently, relatively lengthy synthetic routes and/or expensive starting materials were required. To address these issues, Hamada proposed the de novo introduction of two of the contiguous stereocenters by a sequence of asymmetric aza‐MIRC aziridination, followed by regioselective nucleophilic ring‐opening.

**Scheme 13:**
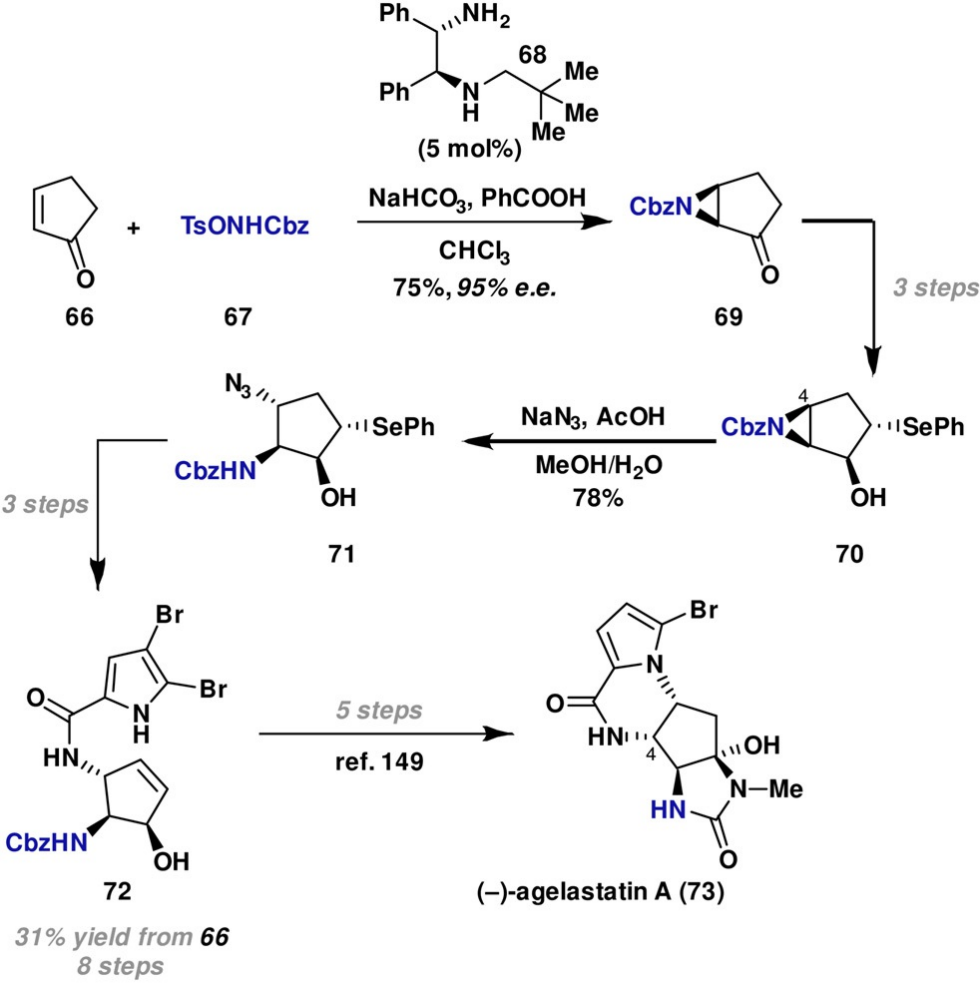
Hamada's formal synthesis of (−)‐agelastatin A (**73**).

The key aza‐MIRC aziridination involved cyclic enone **66** and TsONHCbz **67** as the aminating agent (Scheme 13). It was found that chiral diamine catalyst **68** was essential for achieving high enantioselectivity, and both benzoic acid and NaHCO_3_ additives were required to prevent decomposition of the aminating agent. Under these conditions, **69** was generated in 75 % yield and 95 % *ee*. From here, a three‐step sequence led to alcohol **70**. Ring opening of aziridine **70** with azide was regioselective, occurring solely at C(4) to provide **71** in 78 % yield. It was postulated that the steric interactions of the hydroxyl group enforce the observed regioselectivity of this process. From **71**, three steps were required to access known intermediate **72**, which can be converted to (−)‐agelastatin A (**73**) in a further five steps following a previously reported procedure.^[149]^

In 2018, Nemoto and co‐workers reported a formal synthesis of (−)‐aurantioclavine (**85**), a biologically active, biosynthetic intermediate of a family of complex polycyclic alkaloids, the communesins.^[155]^ The fused tricyclic indole core is a common feature of several alkaloids and so its preparation has been of interest to the synthetic community. It was envisaged that an organocatalytic electrophilic aziridination could be used to install the key C(7) stereocenter of (−)‐aurantioclavine (**85**) prior to Pd‐catalyzed construction of the indole unit. Compared to other approaches, this is one of the only examples where the C(7) benzylic stereocenter is installed without the aid of a metal catalyst.[^156^, ^157^, ^158^]

**Scheme 14:**
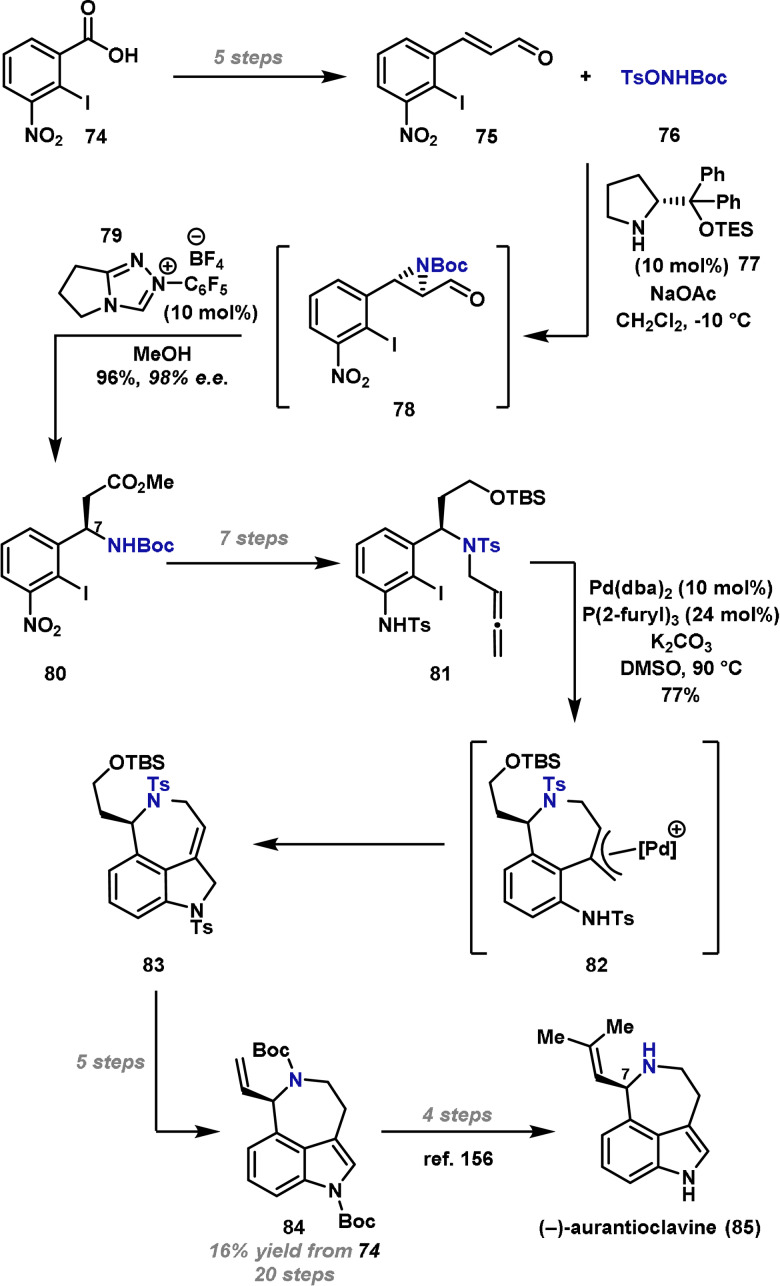
Nemoto's formal synthesis of (−)‐aurantioclavine (**85**). Pd(dba)_2_=bis(dibenzylideneacetone)palladium(0), TBS=*tert*‐butyl(dimethyl)silyl, TES=triethylsilyl.

Enal **75** was prepared in five steps from commercially available 2‐iodo‐3‐nitrobenzoic acid (**74**) (Scheme 14). The organocatalytic asymmetric aziridination was then investigated, and it was found that the combination of proline‐derived organocatalyst **77** and commercially available TsONHBoc **76** was suitable for generating target aziridine **78**. This product was not isolated and, instead, was immediately treated with triazolium salt **79** and MeOH to give β‐aminated ester **80** in 96 % yield and 98 % *ee*. From **80**, seven steps were required to synthesize cascade substrate **81**, as required for construction of the indole subunit. For this process, Pd^0^‐catalyzed conditions were used to effect Heck‐type reaction of the aryl iodide with the allene. This generates π‐allyl intermediate **82**, which can then undergo C−N bond formation to provide **83**, a process that occurred in excellent yield. A further five steps were required to advance **83** to known intermediate **84**, which can be converted to (−)‐aurantioclavine (**85**) in an additional four steps.^[156]^

Although longer than other syntheses,[^156^, ^157^, ^158^, ^159^, ^160^] Nemoto's approach is notable for the aziridinative conversion of **75** to **80**. It is important to highlight that this process transfers the oxidation level, such that acrylate products can be accessed from the enal starting material which is required for the aziridination step. It can be envisaged that adapting this two‐step, one‐pot procedure would allow access to other β‐aminated acrylates and related species at the same oxidation level.

The examples highlighted in this section demonstrate the utility of electrophilic aminating agents in aziridination processes. Importantly, the installation of contiguous stereocenters can be achieved in one or two steps, often from cheap, achiral and commercially available starting materials. The key feature is the use of ambiphilic reagents of type **67** and **76**, and these have found wider application in the development of metal‐free C−N bond formations (see Sections 4 and 6). Additionally, as will be discussed in the next section, reagents **67** and **76** have been integrated into metal‐catalyzed C−N bond formations. On a cautionary note, despite being used on large scale,[^161^, ^162^] care should be taken when handling electrophilic hydroxylamine‐based reagents, as specific variants can be unstable.

## 4 Transition‐Metal‐Catalyzed Processes

Transition‐metal‐promoted C−N bond‐forming reactions are the foremost strategy for the synthesis of amines and their derivatives. The most popular approaches in the context of total synthesis are the Buchwald–Hartwig and Ullmann–Goldberg reactions, which typically involve the reaction of an amine or amide nucleophile with an electrophilic aryl halide in the presence of a palladium or copper catalyst.[^163^, ^164^, ^165^, ^166^, ^167^, ^168^] These methods offer unrivalled flexibility for the construction of aryl C−N bonds. Nevertheless, umpoled strategies have also emerged that allow the cross‐coupling of electrophilic aminating agents with aryl nucleophiles or aryl C−H bonds.[^169^, ^170^, ^171^, ^172^, ^173^, ^174^, ^175^, ^176^] Such processes are valuable as they offer complementary substrate scope and, in some cases, milder reaction conditions.

### 4.1 Aza‐Heck Reaction

It is perhaps in the area of alkene functionalization that transition‐metal‐catalyzed reactions with electrophilic aminating agents are most prolific. Within this context, aza‐Heck reactions represent a key emerging technology;^[177]^ here, an N‐based unit is exploited as the initiating motif, and an alkene functions as the formal nucleophile. Processes of this type are complementary to intramolecular aza‐Wacker reactions, which involve the Pd‐catalyzed oxidative cyclization of an NH nucleophile with an alkene. This is a conceptually appealing method for accessing N‐heterocycles and has been developed extensively.^[178]^ Nevertheless, limitations remain, including the requirement for relatively acidic NH nucleophiles, and a low tolerance to sterically encumbered alkenes. Additionally, versatile chiral P‐based ligands are not well tolerated because of the requirement for an external oxidant, and this limits the applicability of the method in asymmetric catalysis.[^178^, ^179^] All of these issues can be addressed by instead using an aza‐Heck approach, and recently developed variants of this chemistry now offer excellent scope.[^180^, ^181^, ^182^, ^183^, ^184^, ^185^, ^186^]

**Scheme 15:**
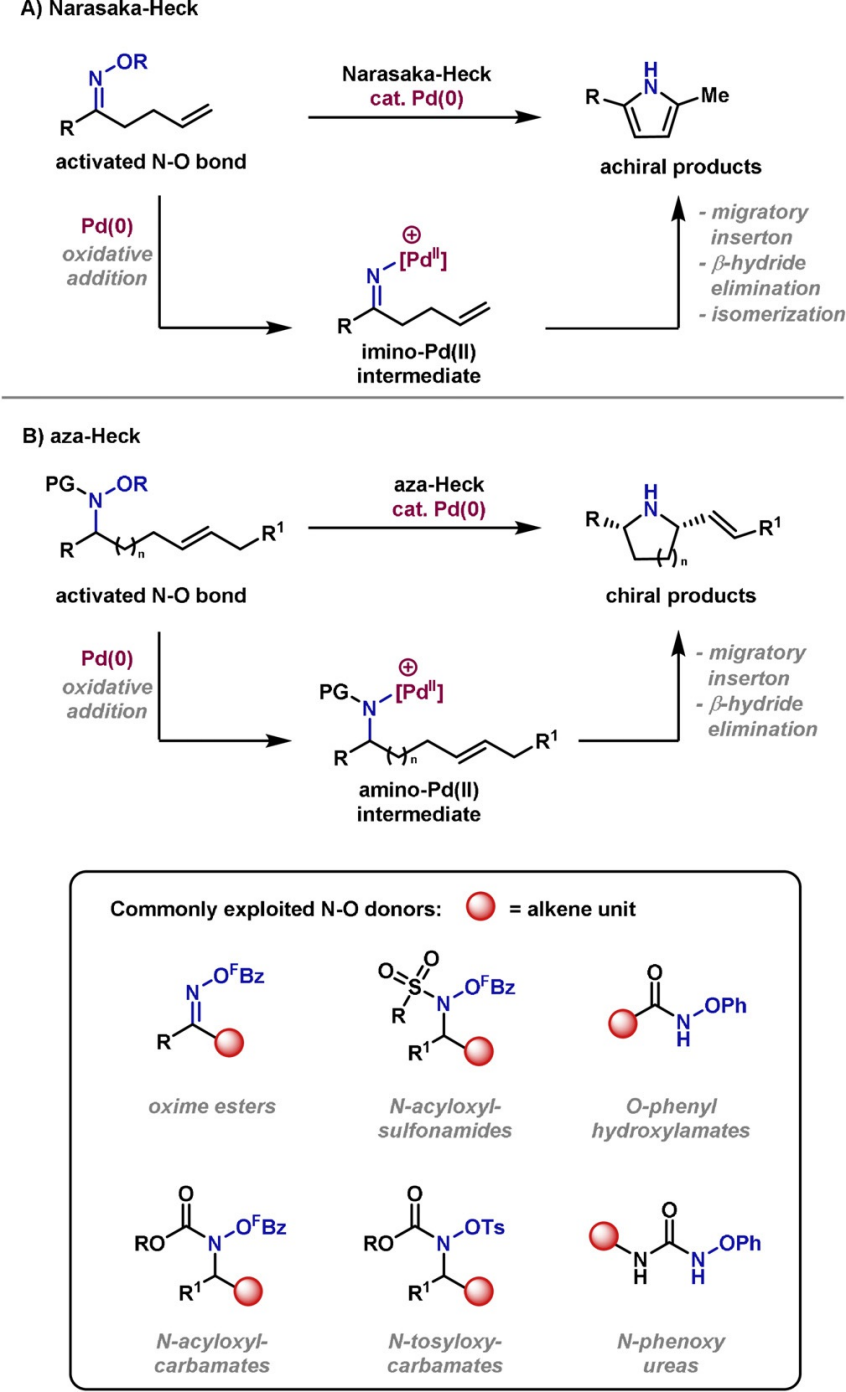
General strategy for Narasaka and aza‐Heck cyclizations and commonly exploited N‐O donors.

The first aza‐Heck reactions were reported in 1999 by Narasaka and co‐workers (Scheme 15 A).^[187]^ In the initial report, oxidative addition of the N−O bond of oxime esters was used to trigger alkene aza‐palladation en route to pyrrole products. This approach has more recently been extended to other classes of N−O bonds. For example, hydroxylamine‐based systems cyclize to provide chiral N‐heterocycles such as pyrrolidines and piperidines (Scheme 15 B).[^180^, ^181^, ^183^, ^184^] In these processes, chiral P‐based ligands can be used to induce enantioselectivity.^[181]^ The most general aza‐Heck reactions employ O‐based leaving groups on nitrogen, such as pentafluorobenzoate (^F^BzO‐), phenolate and tosylate.[^181^, ^182^, ^183^]

**Scheme 16:**
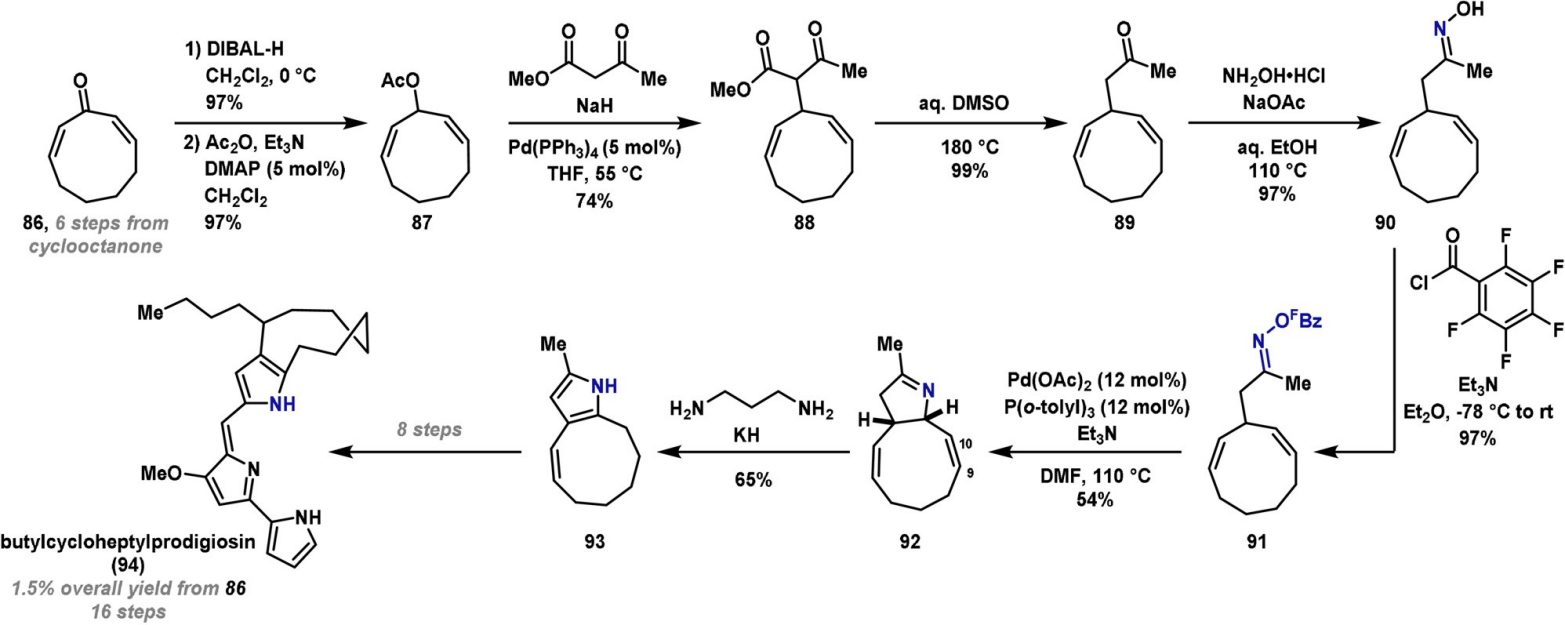
Fürstner's synthesis of butylcycloheptylprodigiosin (**94**). DIBAL‐H=diisobutylaluminum hydride.

In 2005, Fürstner reported the first synthesis of butylcycloheptylprodigiosin (**94**), a purported natural product that has been the source of controversy (Scheme 16).[^188^, ^189^, ^190^] Butylcycloheptylprodigiosin (**94**) consists of a nine‐membered ring fused to a pyrrole, and it was proposed that this key component could be constructed using the Narasaka variant of the aza‐Heck reaction. The synthesis of (*Z,Z*)‐cyclononadienone **86** was carried out in six steps from commercially available cycloctanone. From here, the medium‐ring was elaborated to **87** in excellent yield by 1,2‐reduction of the dienone and subsequent O‐acetylation. Tsuji–Trost reaction with methyl acetoacetate gave **88**, which underwent Krapcho decarboxylation to provide **89**. A typical two‐step sequence, involving conversion to oxime **90** and O‐acylation with pentafluorobenzoyl chloride, provided aza‐Heck precursor **91**. Because at this stage, available aza‐Heck protocols were relatively inefficient, two equivalent alkene units were specifically incorporated into system **91** to enhance cyclization efficiency. Under standard Heck‐like conditions, cyclization generated pyrroline **92** in 54 % yield, and the typical pyrrole product was not observed. This outcome can be rationalized by the *syn*‐stereospecific nature of the migratory insertion and β‐hydride elimination steps, which generates the C(9)–C(10) alkene double bond. Exposure of **92** to potassium 3‐aminopropylamide isomerized the C(9)–C(10) alkene double bond to generate the desired pyrrole product **93** in good yield. From here, a further eight steps were required to advance **93** to butylcycloheptylprodigiosin (**94**).

A more concise synthesis of butylcycloheptylprodigiosin (**94**) has since been reported.^[191]^ Nevertheless, Fürstner's synthesis remains significant within the context of aza‐Heck cyclizations, and is also instructive from a strategy viewpoint. The de novo installation of a pyrrole unit onto a complex, strained ring system demonstrates the value of the aza‐Heck method for the construction of heterocycles at a late stage.

**Scheme 17:**
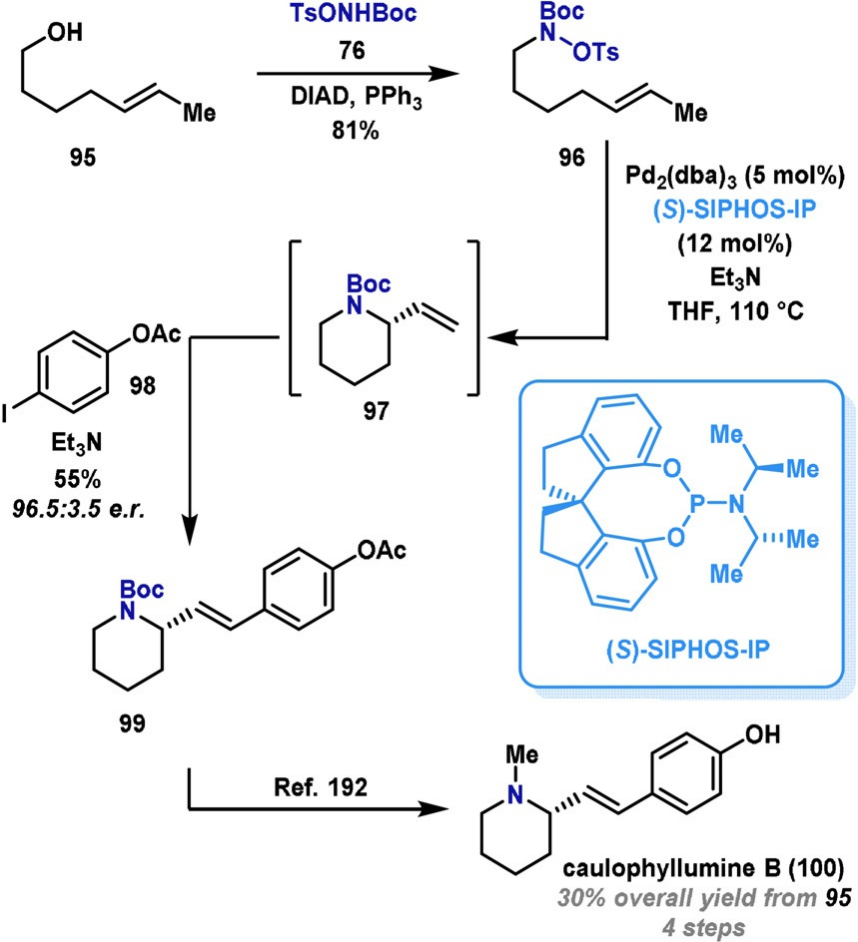
Bower's synthesis of caulophyllumine B (**100**). DIAD=diisopropyl azodicarboxylate, e.r.=enantiomeric ratio, (*S*)‐SIPHOS‐IP=*N*‐dimethyl‐[(*S*)‐1,1′‐spirobiindane‐7,7′‐diyl]phosphoramidite.

Recent years have seen significant methodology development within the area of aza‐Heck chemistry, and this has provided efficient methods for accessing other classes of N‐heterocycles. In particular, enantioselective aza‐Heck cyclizations of *N*‐(tosyloxy)carbamates provide a powerful method for preparing α‐substituted pyrrolidines and piperidines.^[181]^ This chemistry has been used by Bower in an enantioselective, four‐step formal synthesis of caulophyllumine B (**100**), a P450 cytochrome metabolite.^[181]^ Note that a previous synthesis by Reddy and Krishna^[192]^ provided caulophyllumine B in fourteen steps. Bower's synthesis commenced with Mitsunobu reaction of (*E*)‐hept‐5‐en‐1‐ol (**95**) and electrophilic aminating agent **76** to generate hydroxylamine **96** (Scheme 17). From here, previously developed enantioselective aza‐Heck conditions were adapted to a tandem process. Using a Pd^0^ catalyst modified with a SPINOL‐derived phosphoramidite ligand, cyclization of **96** to **97** occurred efficiently. **97** was not isolated, and instead the residual Pd^0^ catalyst was harnessed for a subsequent Heck reaction with aryl iodide **98**. This two‐step/one‐pot catalytic protocol gave piperidine **99** in excellent yield and enantioselectivity. Following Reddy and Krishna's reported procedure, concomitant N‐Boc reduction and ester removal provided caulophyllumine B (**100**) in 30 % overall yield.^[192]^

The syntheses highlighted in this section demonstrate that electrophilic oximes and hydroxylamines can engage with Pd catalysts in redox‐type processes. Following reaction with a pendant alkene, this allows the construction of a range of substituted N‐heterocyclic products. Here, the N−O bond of the aminating agent is analogous to the C−X (X=halide, OTf) bond used in conventional Heck reactions, and, as a result, the fundamental mechanistic steps are the same.^[193]^ Through judicious use of chiral ligand, asymmetric processes can also be realized. Interestingly, bulky electron‐rich phosphine ligands typically used in conventional Heck processes are not usually effective for aza variants, where instead electron‐poor P‐based ligands are optimal. The use of the conventional Heck reaction in total synthesis is well established;[^194^, ^195^, ^196^, ^197^] however, there are relatively few examples of the use of aza variants. This is likely to change as the methodologies become more sophisticated.

### 4.2 Copper‐Catalyzed Hydroamination

**Scheme 18:**
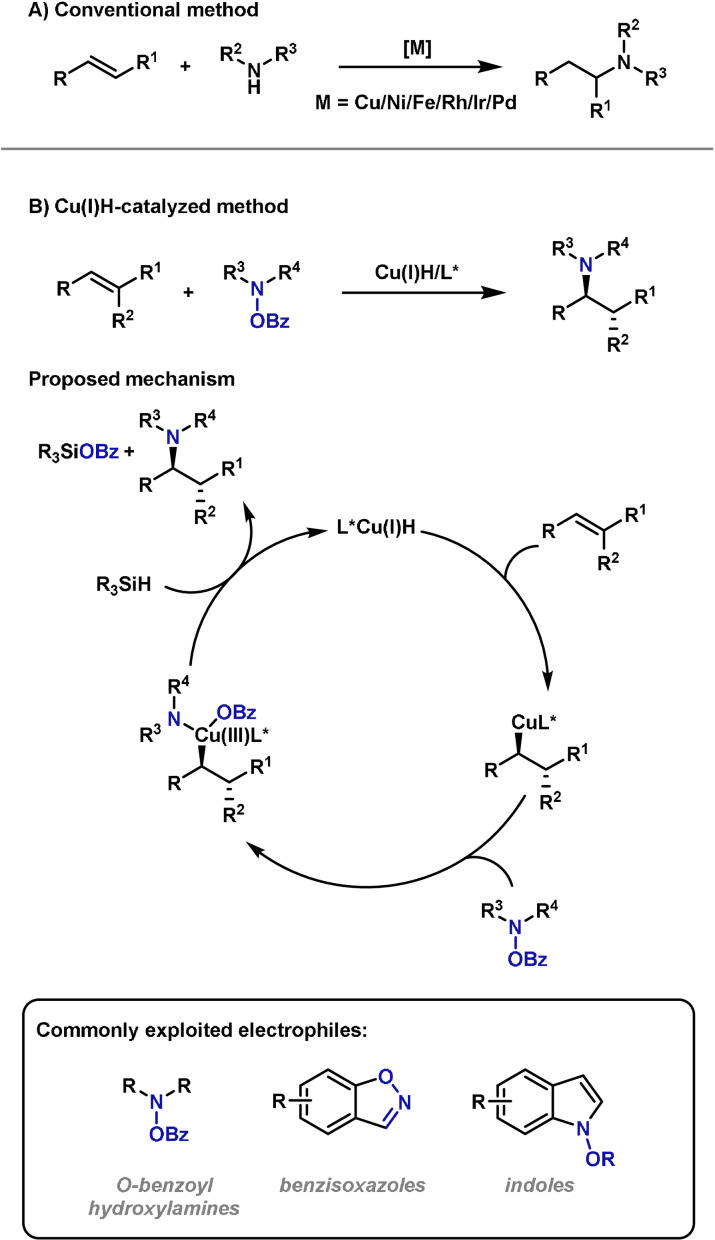
Methods for hydroamination and the proposed mechanism of Cu‐H based variants.

Hydroamination of alkenes and alkynes is a powerful strategy for the formation of C−N bonds. Such processes are complementary to aza‐Heck reactions because the product is generated at a lower unsaturation level. A variety of strategies have emerged for effecting alkene and alkyne hydroaminations, including metal‐[^198^, ^199^, ^200^, ^201^, ^202^, ^203^, ^204^, ^205^, ^206^, ^207^, ^208^, ^209^, ^210^, ^211^] and Bronsted acid catalyzed[^212^, ^213^, ^214^] methods, radical‐mediated processes[^215^, ^216^, ^217^, ^218^, ^219^] and pericyclic‐based methods.[^220^, ^221^] Conventional hydroamination protocols are designed to enable the addition of an amine N−H bond across an alkene or alkyne π‐bond. However, this approach is often limited to activated alkenes and alkynes, and generally suffers from poor enantioinduction in asymmetric processes (Scheme 18 A).[^222^, ^223^, ^224^, ^225^] To address these issues, electrophilic hydroxylamine units have been harnessed in combination with a reductant. This approach allows the hydroamination of nucleophilic non‐activated alkenes and is also well suited to asymmetric transformations. Additionally, the regiochemical outcome of the process (Markovnikov vs. anti‐Markovnikov) is often opposite to that obtained using conventional protocols. Perhaps the most notable class of process in this field are Cu^I^H‐catalyzed hydroaminations pioneered by Miura^[226]^ and Buchwald (Scheme 18 B).^[227]^ In these processes, the alkene undergoes insertion into a LCu^I^–H species to form an alkylcopper complex. Oxidative addition of the electrophilic aminating agent is then followed by reductive elimination to deliver the product with the new C−N bond. The Cu^I^ hydride catalyst is regenerated by transmetalation with an external hydride source. Since the initial reports, copper‐catalyzed hydroaminations of this type have rapidly developed and now offer wide scope.[^228^, ^229^, ^230^, ^231^, ^232^, ^233^, ^234^, ^235^, ^236^, ^237^, ^238^] The most commonly exploited electrophilic aminating agents are *O*‐benzoylhydroxylamine derivatives. More recently, processes that exploit distinct classes of electrophilic nitrogen sources such as benzisoxazoles^[233]^ have emerged.

**Scheme 19:**
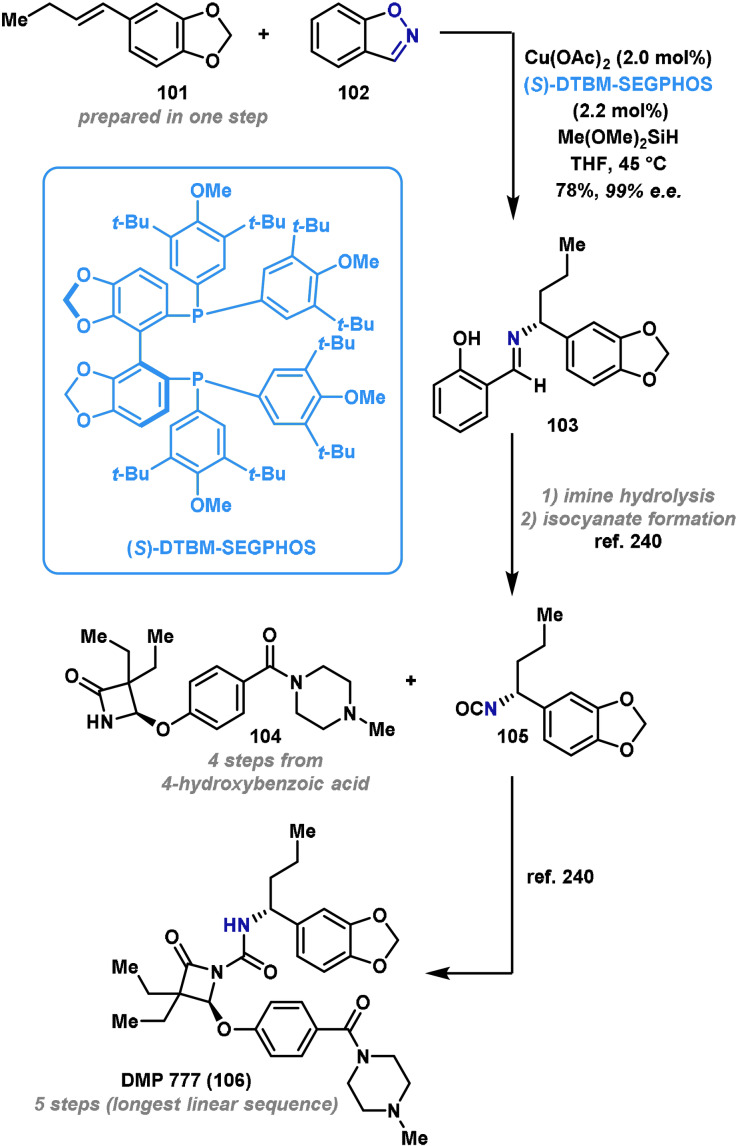
Buchwald's application of Cu^I^H hydroamination to the synthesis of DMP 777 (**106**). (*S*)‐DBTM‐SEGPHOS=(*S*)‐(+)‐5,5‐bis[di(3,5‐di‐*tert*‐butyl‐4‐methoxyphenyl)phosphino]‐4,4‐bi‐1,3‐benzodioxole.

In 2018, Buchwald reported the application of copper‐catalyzed hydroamination in the preparation of **105**, a key intermediate in the synthesis of DMP 777 (**106**).^[233]^ DMP 777 (**106**) is a known inhibitor of leukocyte elastase, and has potential for treatment of cystic fibrosis and rheumatoid arthritis.^[239]^ Previous syntheses of **105** have relied on the use of expensive chiral starting materials or enzymatic resolution to introduce the required stereocenter.^[240]^ Buchwald's approach allows the introduction of the key amino‐bearing stereocenter from cheap, achiral starting materials by employing an asymmetric copper‐catalyzed hydroamination reaction (Scheme 19).

Compound **103** was prepared by subjecting alkene **101** to the Cu^I^H hydroamination conditions, in the presence of the chiral ligand (*S*)‐DTBM‐SEGPHOS. In this particular case, 1,2‐benzisoxazole (**102**) was selected as the aminating agent and this provided the Schiff base product **103** in excellent yield and enantioselectivity. From here, hydrolysis of the imine gave the desired chiral amine, which was advanced to the corresponding isocyanate, **105**. This can be coupled with known amide **104** to generate DMP 777 (**106**) in excellent yield.^[240]^

**Scheme 20:**
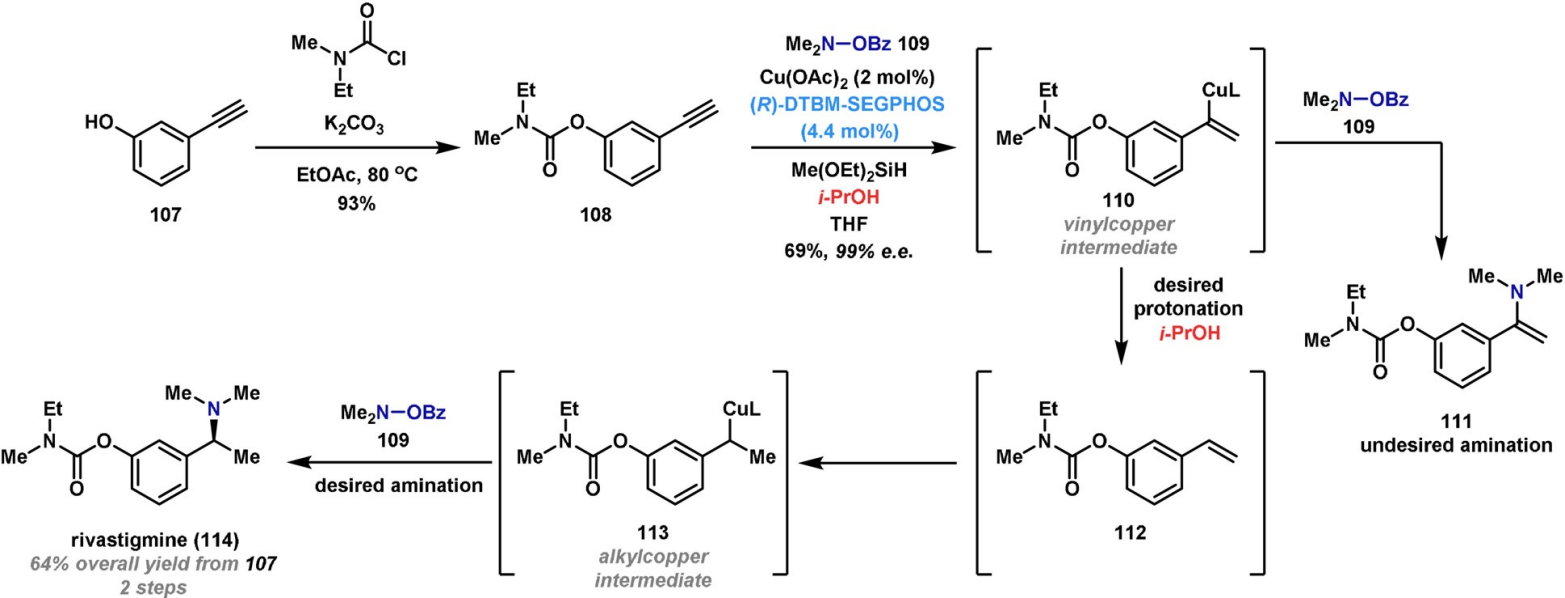
Buchwald's synthesis of rivastigmine (**114**).

To demonstrate the applicability of Cu^I^H‐catalyzed hydroamination to other types of π‐unsaturated systems, Buchwald reported a succinct two‐step synthesis of rivastigmine (**114**), a drug compound used for the treatment of Alzheimer's and Parkinson's disease (Scheme 20).^[230]^ The synthesis commenced with the formation of carbamate **108** by addition of *N*‐ethyl‐*N*‐methylcarbamoyl chloride to commercially available 3‐hydroxyphenylacetylene (**107**). From here, the amino‐bearing stereocenter was introduced by a tandem process involving Cu^I^H‐catalyzed reduction of the alkyne and hydroamination of the resulting alkene. The reduction step required the addition of *i*‐PrOH, which is postulated to protonate vinyl copper species **110**, and thereby prevent formation of enamine **111**. The resulting alkene **112** then engages in another CuH‐catalyzed hydroamination event via the alkylcopper species **113** to provide rivastigmine (**114**) in excellent yield and enantioselectivity.

The syntheses outlined in this section show that electrophilic aminating agents can combine with transition metals in redox‐type processes to provide new C−N bonds. The two process classes that have been discussed both involve aminations of unsaturated carbon‐based units (e.g. alkenes), but it is important to recognize that they are mechanistically distinct. Aza‐Heck reactions initiate on the amino partner, whereas CuH‐catalyzed hydroaminations initiate on the alkene or alkyne. Importantly, both process classes can engage non‐polarized alkenes in an enantioselective manner. The flexibility and control provided by these methods means that they are likely to see increasing use in total synthesis. It should be noted that recently developed enantioselective photocatalytic hydroaminations with sulfonamides offer a powerful alternative approach for effecting olefin hydroamination.^[241]^

## 5 Electrophilic N‐Centered Radicals

Processes involving carbon‐centered radicals offer unique reactivity and functional group tolerance.^[242]^ In recent years, interest in the use of heteroatom‐centered radicals has gained traction, and within this area, the use of nitrogen‐based radicals has been extensively investigated. This has resulted in a range of interesting methods for the construction of C−N bonds.[^243^, ^244^, ^245^]

**Scheme 21:**
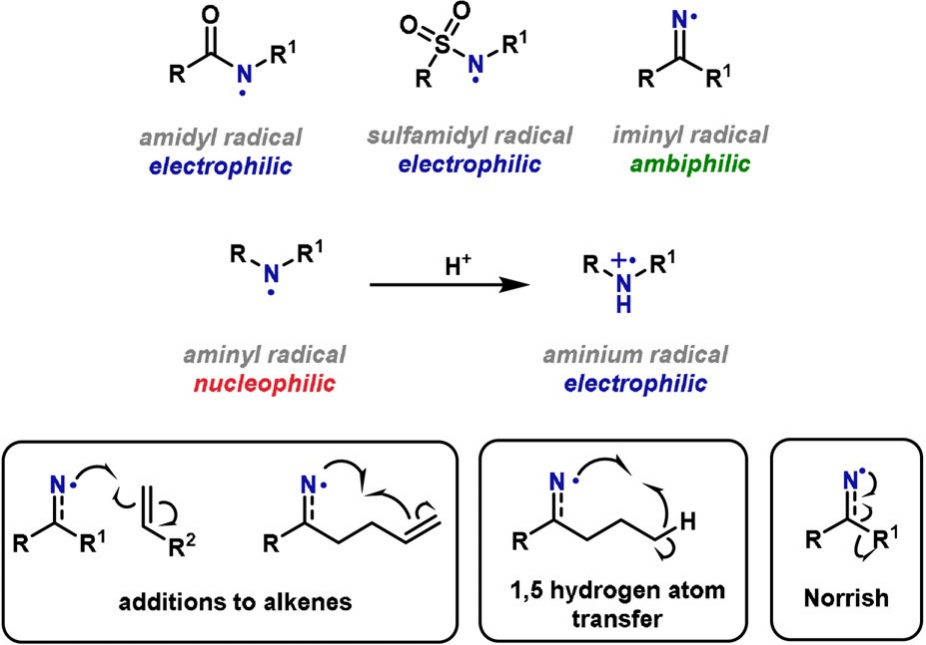
Nitrogen‐centered radicals.

A well‐established method for the generation of nitrogen‐centered radicals is by photochemical or thermal dissociation of an N−X bond, where X is a heteroatom‐based unit or a halogen. Conventionally, this approach has been hampered by the requirement for toxic radical initiators, high temperatures and/or high‐energy UV irradiation. The advent of photocatalytic methods has enabled milder protocols for the generation of nitrogen radicals in a controlled manner, and this bodes well for future developments in this area.[^246^, ^247^] The properties of nitrogen‐centered radicals are strongly influenced by the specific N‐substituents. With the exception of iminyl radicals, which are usually considered ambiphilic, most N‐centered radicals can be classed as either electrophilic or nucleophilic (Scheme 21).[^248^, ^249^, ^250^] Electrophilic nitrogen radicals include amidyl, sulfamidyl and aminium variants, whereas aminyl radicals are nucleophilic. It is also important to note that aminyl radicals can be converted to aminium species by protonation. All radicals can be generated from readily available functional groups and as a result, are well suited to application in total synthesis.

Electrophilic nitrogen radicals engage in a multitude of processes, most notably intramolecular and intermolecular additions to alkenes. Under photocatalytic conditions, primary and secondary alkyl amines can be converted directly to the corresponding aminium radical, and these can be used for hydroaminations of non‐activated alkenes.[^251^, ^252^] Related radical‐based processes that use electrophilic nitrogen sources enable, for example, alkene aminochlorination reactions.[^253^, ^254^] Less commonly, electrophilic nitrogen radicals are employed in hydrogen‐atom‐transfer reactions and fragmentations related to the classical Norrish I and II reactions.[^255^, ^256^] It is important to note that, due to differences in philicity, not all electrophilic nitrogen radicals are amenable to all types of transformations. A distinct highlight is that electrophilic nitrogen radicals can engage in the functionalization of typically unreactive bonds, and this feature can be harnessed for the synthesis of complex N‐heterocycles.

### 5.1 Amidyl Radicals

Amidyl radicals have found extensive use in synthesis due to their high reactivity, functional group tolerance and profoundly electrophilic character.[^248^, ^257^, ^258^, ^259^, ^260^, ^261^, ^262^] However, their high reactivity presents challenges because competitive intramolecular H‐atom abstraction reactions can often occur in preference to the target C−N bond formation.[^256^, ^263^] Despite this, amidyl radicals provide a popular platform for the synthesis of polyheterocycles. Until recently, amidyl radicals were typically prepared by homolysis of an N−O or N–halogen bond under harsh reaction conditions. However, there are now a number of milder photocatalytic methods for this transformation.[^246^, ^264^, ^265^] The most common precursors used in total synthesis are hydroxylamine derivatives,^[266]^ thiosemicarbazides^[267]^ and thiosemicarbazones.^[268]^ All are easily prepared, and hydroxylamine‐based systems are particularly advantageous as the N−O bond is weak.

**Scheme 22:**
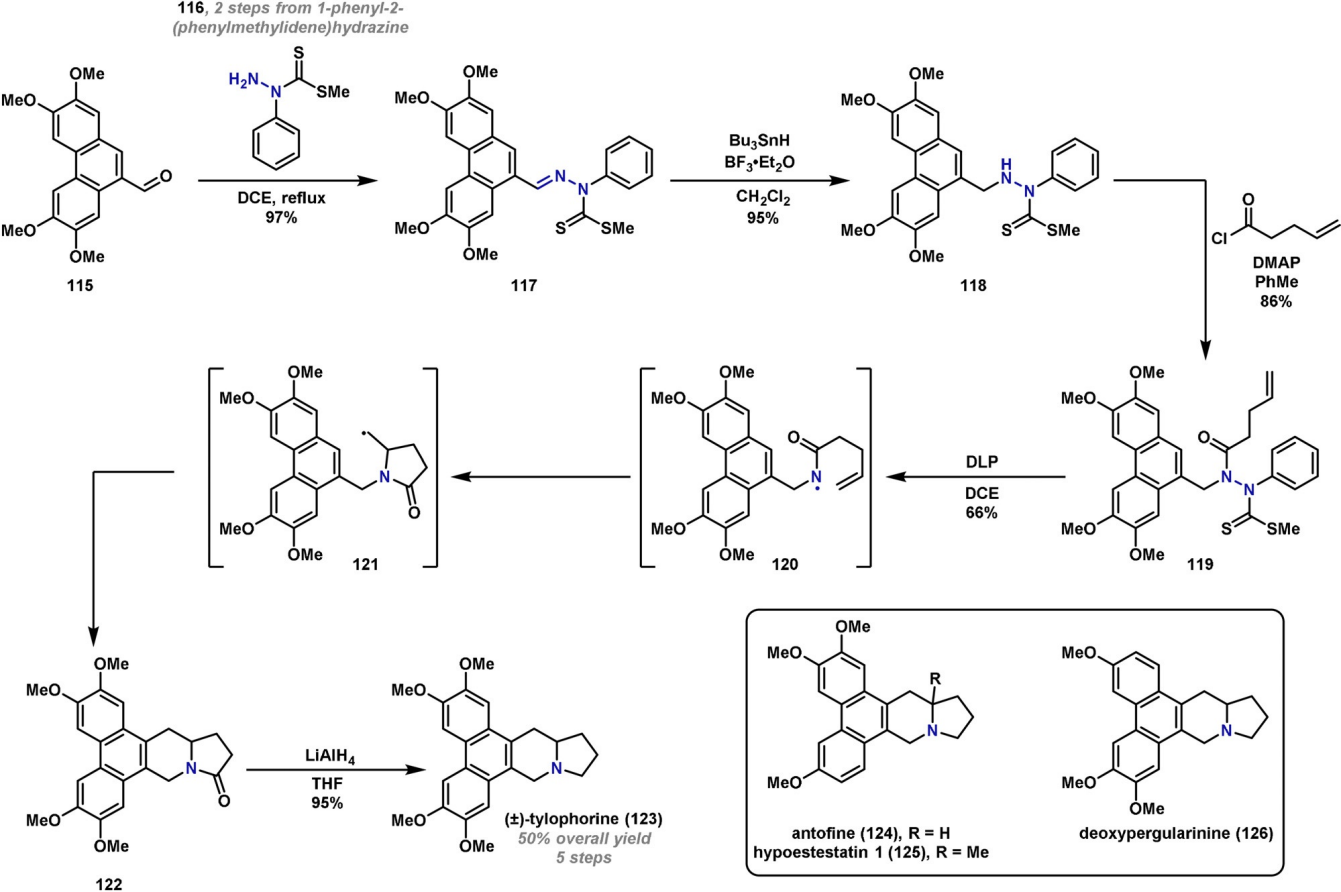
Wang's synthesis of (±)‐tylophorine (**123**). DCE=1,2‐dichloroethane, DLP=dilauroyl peroxide.

In 2013, Wang reported a concise synthesis of several phenanthroindolizidine alkaloids, whereby an unusual amidyl radical cascade/rearrangement sequence was employed as a key step.^[269]^ There are over sixty reported alkaloids in this family,^[270]^ and many are known to have potent bioactivities. As a result, there have been several reported syntheses; however, very few allow access to a wide array of these alkaloids.[^271^, ^272^] In the Wang approach, a radical cascade provides rapid access to the common polyheterocyclic core, thus enabling a general route to many members of the alkaloid family. In this process, 5‐*exo* cyclization of electrophilic amidyl radical **120** with the internal olefin is followed by 6‐*endo* cyclization of the resulting carbon‐centered radical **121**. For the purposes of this review, only the synthesis of (±)‐tylophorine (**123**) will be discussed in detail, but it is worth noting that the strategy has been used for the synthesis of several related alkaloids, including antofine (**124**), hypoestestatin 1 (**125**) and deoxypergularinine (**126**) (Scheme 22). The synthesis of (±)‐tylophorine (**123**) commenced with preparation of amidyl radical precursor **119**. Phenanthrene carbaldehyde **115** underwent condensation with 1‐phenylhydrazinecarbodithioate **116**, which is available in two steps^[273]^ to give **117** in excellent yield. Lewis acid mediated hydrostannation gave hydrazide **118**, which was acetylated with pent‐4‐enoyl chloride to give the electrophilic radical precursor **119**. From here, addition of a stoichiometric quantity of dilauroyl peroxide (DLP), a well‐known radical initiator and oxidant, led to the formation of the key amidyl radical **120**. This engaged in the expected radical cascade to give amide **122** in good yield. Finally, amide reduction generated (±)‐tylophorine (**123**) in just five steps from **115**. Although rapid, an issue with this strategy is that it is not well suited to an asymmetric synthesis. This aspect has since been addressed through alternative syntheses developed by several groups.[^274^, ^275^, ^276^]

**Scheme 23:**
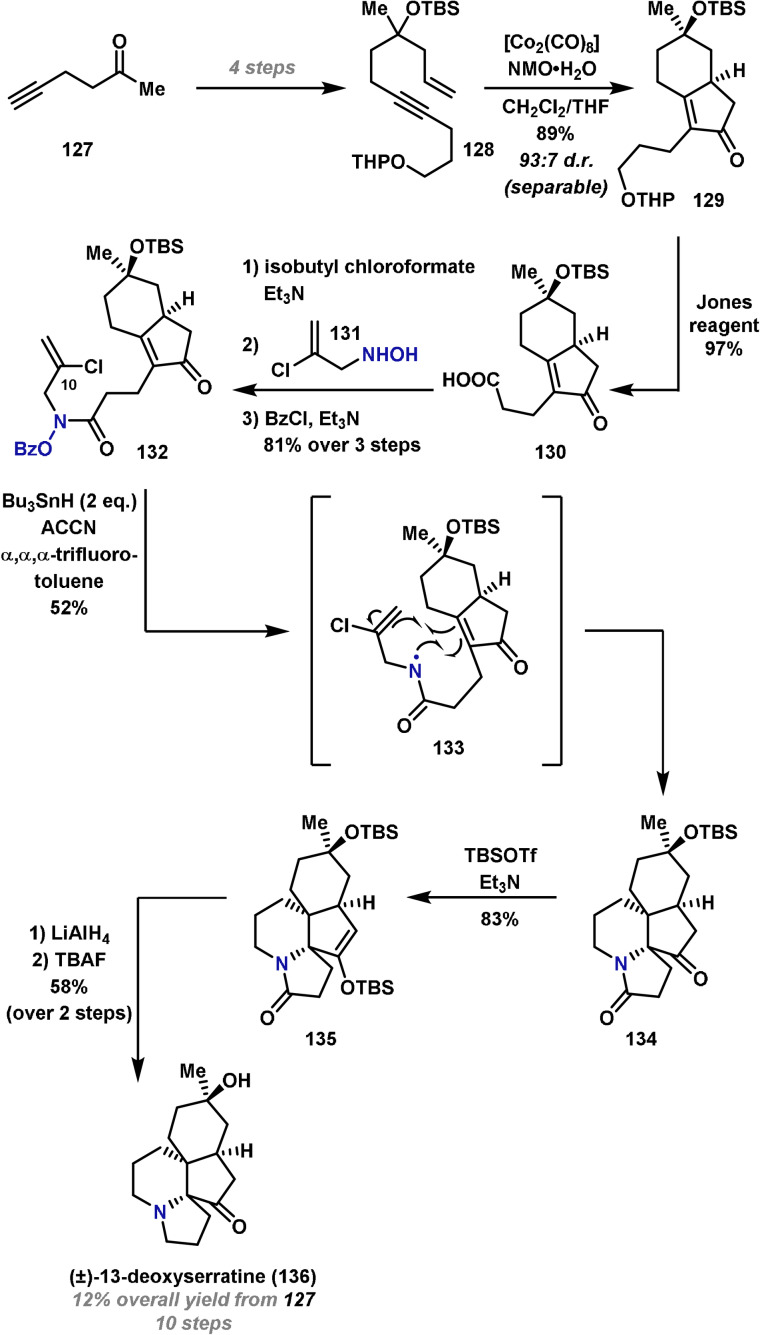
Zard's synthesis of (±)‐13‐deoxyserratine (**136**). ACCN=1,1′‐azobis(cyanocyclohexane), NMO=*N*‐methylmorpholine *N*‐oxide, TBAF=tetrabutylammonium fluoride, THP=tetrahydropyran.

Amidyl radicals have been exploited in the design of other types of polycyclizations cascades. A notable example was reported by Zard and co‐workers in 2002 in the first synthesis of (±)‐13‐deoxyserratine (**136**).^[277]^ Their route commenced with the preparation of the Pauson–Khand precursor **128**, which was synthesized in four steps from commercially available 5‐hexyn‐2‐one (**127**) (Scheme 23). Treatment of **128** with [Co_2_(CO)_8_] and NMO initiated the Pauson–Khand reaction and gave the desired bicyclo[4.3.0]nonenone **129** in excellent yield and diastereoselectivity. Oxidation of the THP‐protected alcohol with Jones reagent gave acid **130** in good yield. Formation of the mixed anhydride, addition of hydroxylamine **131** and benzoyl activation of the OH unit gave radical precursor **132**. The key cascade was initiated by the addition of Bu_3_SnH and 1,1′‐azobis(cyanocyclohexane) (ACCN). The amidyl radical **133** generated in this manner attacked the less hindered, convex face of the enone, triggering 6‐*endo* cyclization of the ensuing carbon‐centered radical. This sequence installs the two adjacent and hindered tetrasubstituted stereocenters of the target molecule. The inclusion of a chlorine atom at C(10) was required to enforce 6‐*endo* (rather than 5‐*exo*) selectivity during the second cyclization. A second equivalent of Bu_3_SnH was therefore required to remove the chlorine atom in situ after the radical cascade, thereby allowing access to **134** in good yield. Finally, protection of the ketone as its silyl enol ether was followed by amide reduction and deprotection to give (±)‐13‐deoxyserratine (**136**). The core structural motif is common in several alkaloids and so a cascade reaction of this type could be exploited in the synthesis of other alkaloids. Previous strategies to install a similar core have required lengthy and low‐yielding synthetic routes.^[278]^

**Scheme 24:**
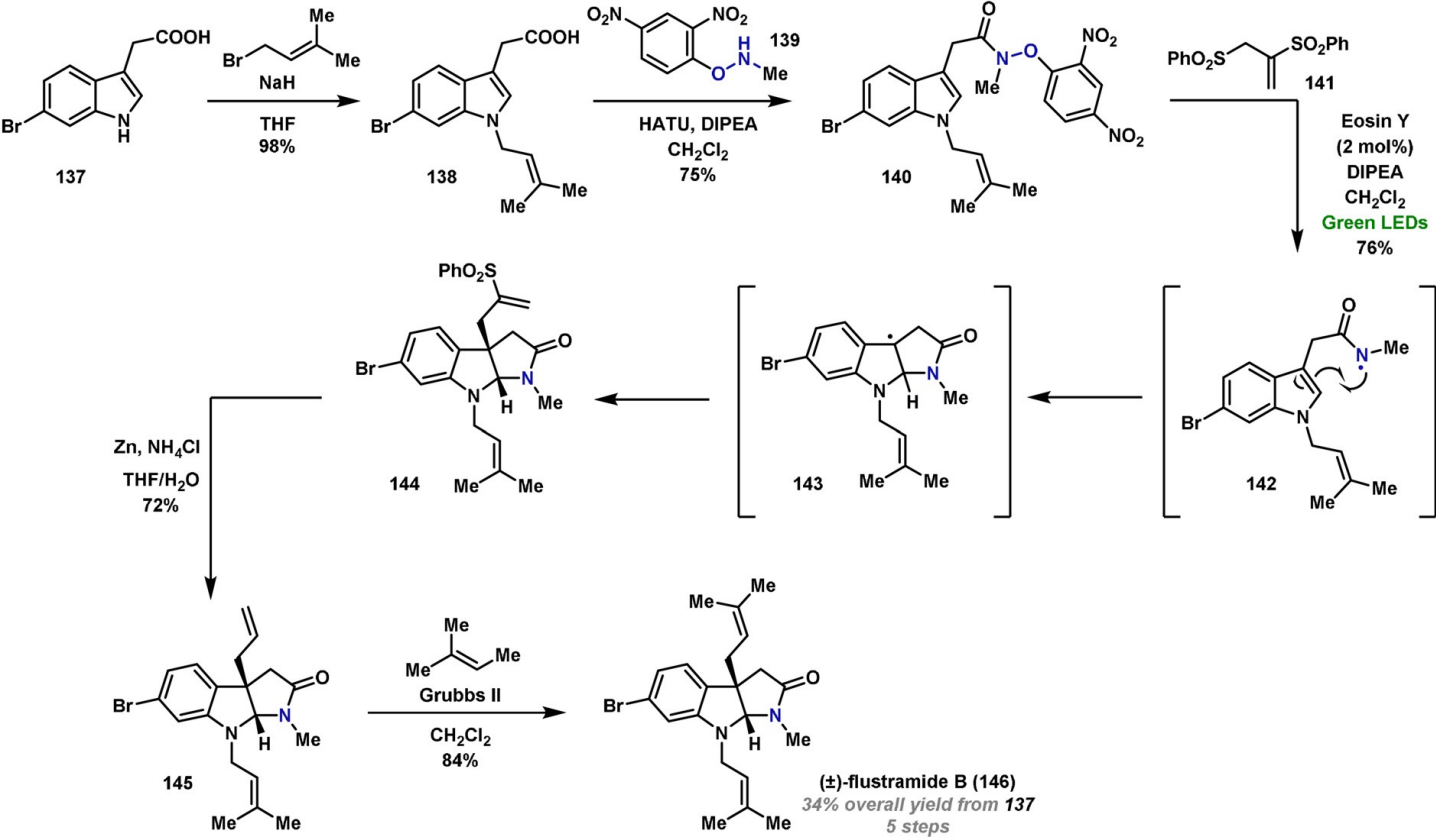
Wang's synthesis of (±)‐flustramide B (**146**).

The field of photoredox chemistry has led to the development of mild photocatalytic methods for the formation of amidyl radicals, and these are likely to find wide use in the design of total synthesis oriented cascades.[^246^, ^265^] An instructive example of the utility of this approach is showcased in Wang's synthesis of (±)‐flustramide B (**146**), a marine alkaloid that has the potential as a muscle relaxant.^[279]^ The synthesis begins with N‐prenylation of commercially available indole **137** to give **138** in excellent yield (Scheme 24). Subsequent amide coupling with **139** gave the electrophilic amidyl radical precursor **140** in good yield. The electron‐poor aryloxy amide was selected as the N−O bond is weak, and this allowed the generation of amidyl radical **142** under mild photocatalytic conditions, in this case using Eosin Y as the photocatalyst. 5‐*endo* cyclization of **142** provided carbon‐centered radical **143**, which was trapped in situ with vinyl sulfone **141** to yield the pyrroloindoline core **144** in good yield. Reduction of the C−S bond of the vinyl sulfone gave **145** and, finally, cross‐metathesis with 2‐methyl‐2‐butene gave (±)‐flustramide B (**146**) in just five steps and in excellent yield. A challenge going forward is to reengineer the key radical‐based cascade to provide an asymmetric synthesis.

### 5.2 Aminyl Radicals

Aminyl radicals are generally considered to be nucleophilic; however, they are often generated from electrophilic sources of nitrogen. As with amidyl radicals, aminyl radicals are also valuable intermediates in the synthesis of polyheterocycles, although in comparison, this area of chemistry is relatively underdeveloped, with only a few examples reported.[^243^, ^280^, ^281^, ^282^, ^283^, ^284^, ^285^] The formation of an aminyl radical is typically carried out from an electrophilic N‐halogen moiety using a radical initiator or harsh photolytic methods,^[280]^ although their generation from the dissociation of N−O^[286]^ and N−S bonds[^287^, ^288^] has also been reported. As these intermediates are less commonly exploited, the development of photocatalytic methods for their formation are not well advanced.

**Scheme 25:**
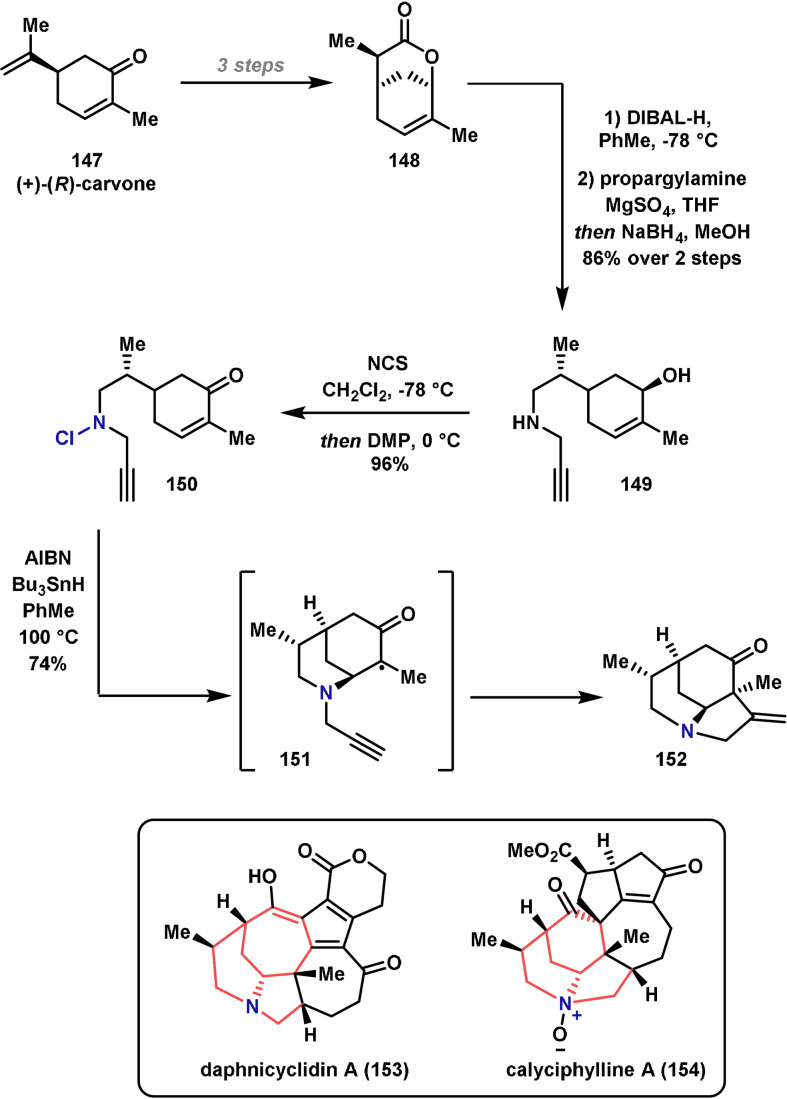
Stockdill's synthesis of the core of the daphnicyclidin (**153**) and calyciphylline (**154**) alkaloids. AIBN=azobisisobutyronitrile, DMP=Dess–Martin periodinane, NCS=*N*‐chlorosuccinimide.

In 2014, Stockdill and co‐workers reported the synthesis of the tertiary amine‐containing polyheterocyclic core of the daphnicyclidin A (**153**) and calyciphylline A (**154**) alkaloids, which are known to exhibit cytotoxicity against murine leukaemia.[^289^, ^290^] Previous syntheses of this core structure have typically involved several synthetic steps, and very few examples have been carried out with complete diastereocontrol.^[291]^ In the Stockdill approach, the polycyclic core is constructed in one step by a cascade cyclization involving an aminyl radical (Scheme 25). The route commenced with the synthesis of bicyclic lactone **148**, prepared in three steps from commercially available (+)‐(*R*)‐carvone **147**. From here, reduction of the lactone to the lactol was followed by reductive amination with propargylamine to give amino alcohol **149** in good yield. The addition of NCS enabled the formation of the required chloroamine and the alcohol was subsequently oxidized with DMP to give cyclization substrate **150**. It was envisaged that homolytic cleavage of the electrophilic N−Cl bond would generate an aminyl radical, which could engage in an intramolecular 6*‐exo* addition to the cyclic enone to give the carbon‐centered radical intermediate **151**. From here, 5‐*exo* cyclization with the pendant alkyne and subsequent hydrogen‐atom abstraction from Bu_3_SnH would generate the desired polycyclic core. Indeed, upon exposure of **150** to AIBN and Bu_3_SnH, the cascade reaction proceeded as expected to generate **152** in excellent yield, and importantly as a single diastereomer. Although this sequence has not so far been employed directly in the synthesis of daphnicyclidin A (**153**) and calyciphylline A (**154**), the studies demonstrate the power of N‐centered radical cascades in reaction design.

The syntheses highlighted in this section outline the use of conventional and photocatalytic methods for the generation of both electrophilic and nucleophilic N‐centered radicals from electrophilic nitrogen sources. Although there are several other classes of N‐centered radicals reported in the literature, these are not as well represented in total synthesis, primarily because mild photocatalytic methods for their generation are underdeveloped. Key issues in this area include competing H‐atom abstraction, which necessitates careful substrate design, and the fact that asymmetric radical‐based processes are still challenging. Nonetheless, the use of N‐centered radicals is one of the most powerful strategies for the construction of densely functionalized, complex polyheterocycles.

## 6 Outlook

The examples highlighted in this review show how electrophilic aminating agents can be used to access N‐containing products via a wide array of mechanistic regimes. Clearly, wider application of these reagents in total synthesis will go hand in hand with further methodology development. It is pertinent therefore at this stage to highlight a selection of recent methodologies that seem well suited to applications in total synthesis.

### 6.1 C−N Bond‐Forming Dearomatizations

**Scheme 26:**
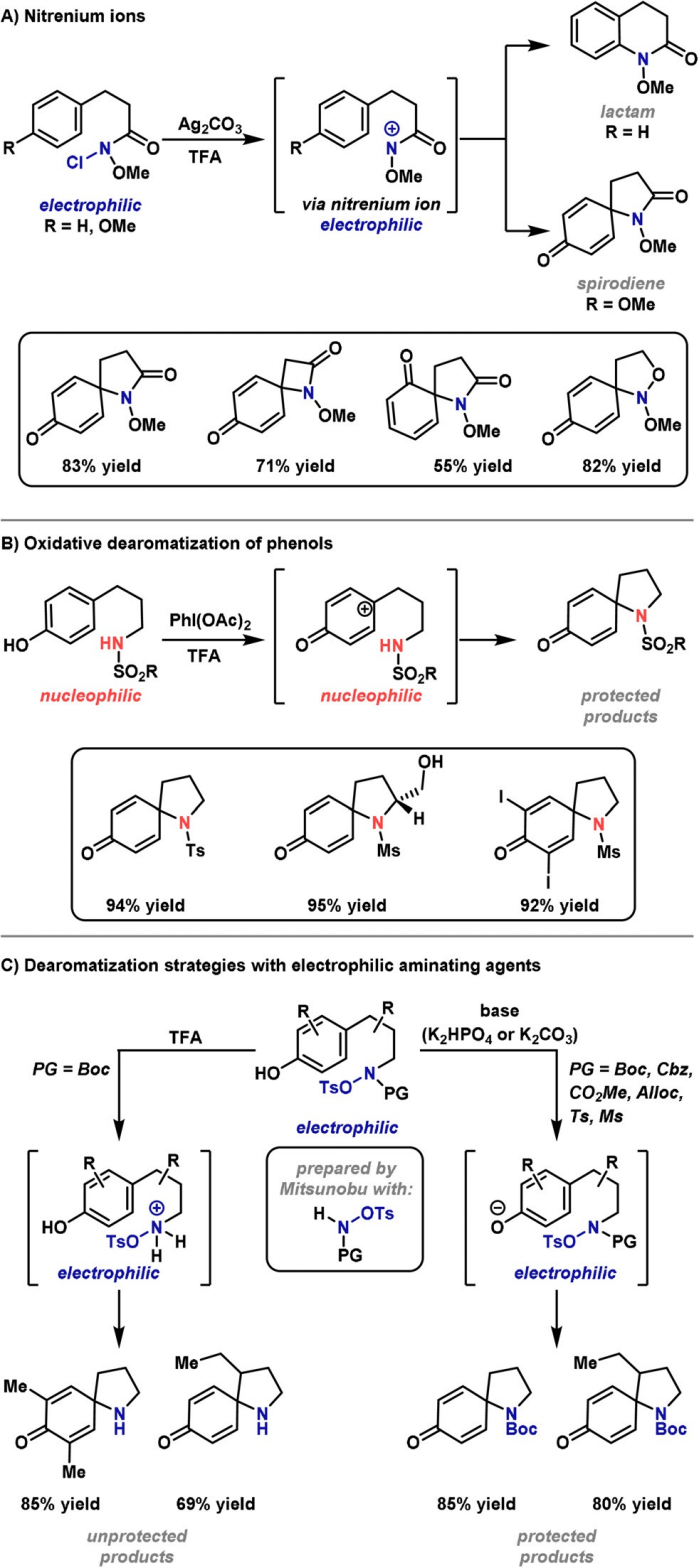
Methods for the synthesis of spirocyclic compounds. Alloc=allyloxycarbonyl, Ms=methanesulfonyl, PG=protecting group.

Until recently, C−N bond‐forming dearomatization reactions were relatively scarce. Classically, processes of this type have been achieved via electrophilic nitrenium ions (Scheme 26 A).[^18^, ^292^] These reactive intermediates can be formed by treatment of an N‐chloroamine with a silver salt.[^18^, ^293^] More recently, hypervalent iodine reagents have emerged as a milder alternative for the in situ preparation of nitrenium ions.^[294]^ These protocols have found application in several syntheses, including the total synthesis of (−)‐swainsonine, (−)‐dysibetaine, (±)‐adalinine and the formal synthesis of (−)‐TAN1251A.[^295^, ^296^, ^297^, ^298^] Although a useful strategy, the highly reactive nitrenium ion requires very specific stabilizing substituents and this severely restricts substrate scope. It is worth noting that in the absence of stabilizing substituents, chlorinated nitrogen centers can participate in an Ag^I^‐promoted Stieglitz rearrangement, a process that has been used elegantly in a total synthesis of (±)‐lycopodine.[^299^, ^300^]

To overcome the aforementioned limitations, Ciufolini and co‐workers have described a conceptually distinct oxidative dearomatization method (Scheme 26 B).[^301^, ^302^, ^303^, ^304^, ^305^, ^306^, ^307^] This method can be rationalized via oxidation of the arene to its corresponding carbocation, which is then trapped by a tethered nitrogen nucleophile. Spirocyclizations of this type have been employed in the total synthesis of several alkaloids, including FR901 483, TAN1251C, (−)‐cylindricine C and (−)‐2‐epicylindricine C, and provide a useful strategy;[^308^, ^309^] however, specific N‐protecting groups are required (esp. sulfonamides), and the requirement for a hypervalent iodine oxidant means that oxidatively sensitive functionality is not well tolerated.

Recently two new complementary C−N bond‐forming dearomatization methods have been reported that exploit hydroxylamine‐based electrophilic aminating agents (Scheme 26 C).^[310]^ In both approaches, the precursor is set up by Mitsunobu alkylation, which, in turn, allows the controlled installation of a stereocenter adjacent to nitrogen. The first method requires substrates bearing an N‐Boc group and occurs under acidic (TFA) conditions. A possible mechanism involves acid‐mediated N‐Boc deprotection to afford an electrophilic nitrogen intermediate. This highly reactive species functions as a potent electrophile such that S_E_Ar‐like attack by the pendant arene leads to unprotected spirocycles. The products retain nucleophilic and electrophilic functionality that can be engaged directly in further bond formations. The second approach provides products where the N‐center is protected and occurs under basic conditions. Notably, the method tolerates a range of protecting groups (e.g. carbamates, sulfonamides) and offers broad scope with respect to the aromatic nucleophile.^[311]^ Compared to prior approaches, these methods are relatively mild and flexible, such that they seem well suited to applications in total synthesis. It is also important to note that certain types of dearomatizing aminations can be achieved under transition‐metal‐catalyzed conditions, and these protocols will also likely find use in total synthesis.[^312^, ^313^, ^314^, ^315^, ^316^]

It is pertinent to highlight that a range of powerful metal‐free *intermolecular* C−N bond‐forming dearomatization processes have been developed recently by Sarlah and co‐workers.[^24^, ^317^, ^318^] In these processes, the light‐promoted cycloaddition of 4‐methyl‐1,2,4‐triazoline‐3,5‐dione (MTAD) with non‐activated arenes is used to generate cycloadducts that can be exploited in further processes. The chemistry has been applied to total syntheses of (+)‐pancrastatin, (+)‐7‐deoxyprancrastatin, (+)‐lycoridine, (+)‐narciclasine and (±)‐conduramine A.[^24^, ^317^, ^318^, ^319^] This area is not discussed in detail here because it has been the subject of a recent review, to which the reader is directed.^[320]^

### 6.2 C‐H Amination Strategies with Electrophilic Radicals

As discussed already, the most commonly exploited methods for aryl C−N bond formation are the Buchwald–Hartwig, Chan–Lam and Ullmann cross‐couplings. These processes offer tremendous utility, but they require prefunctionalization of the C‐based reaction partner, and in certain cases this can be problematic. Consequently, alternative processes have been developed that enable the formation of aryl amines by C‐H amination. This area has seen rapid growth and there are now a variety of methods available;[^321^, ^322^, ^323^, ^324^, ^325^, ^326^, ^327^, ^328^, ^329^, ^330^, ^331^] several of these involve electrophilic nitrogen sources, and these can operate via closed‐^[310]^ or open‐shell (radical) pathways. One appealing strategy that has garnered interest is the use of aminium radicals in C‐H amination processes. As discussed previously, aminium radicals can be formed either by dissociation of an N−X (X=Hal) bond and protonation of the resulting aminyl radical, or by direct photocatalytic methods.

**Scheme 27:**
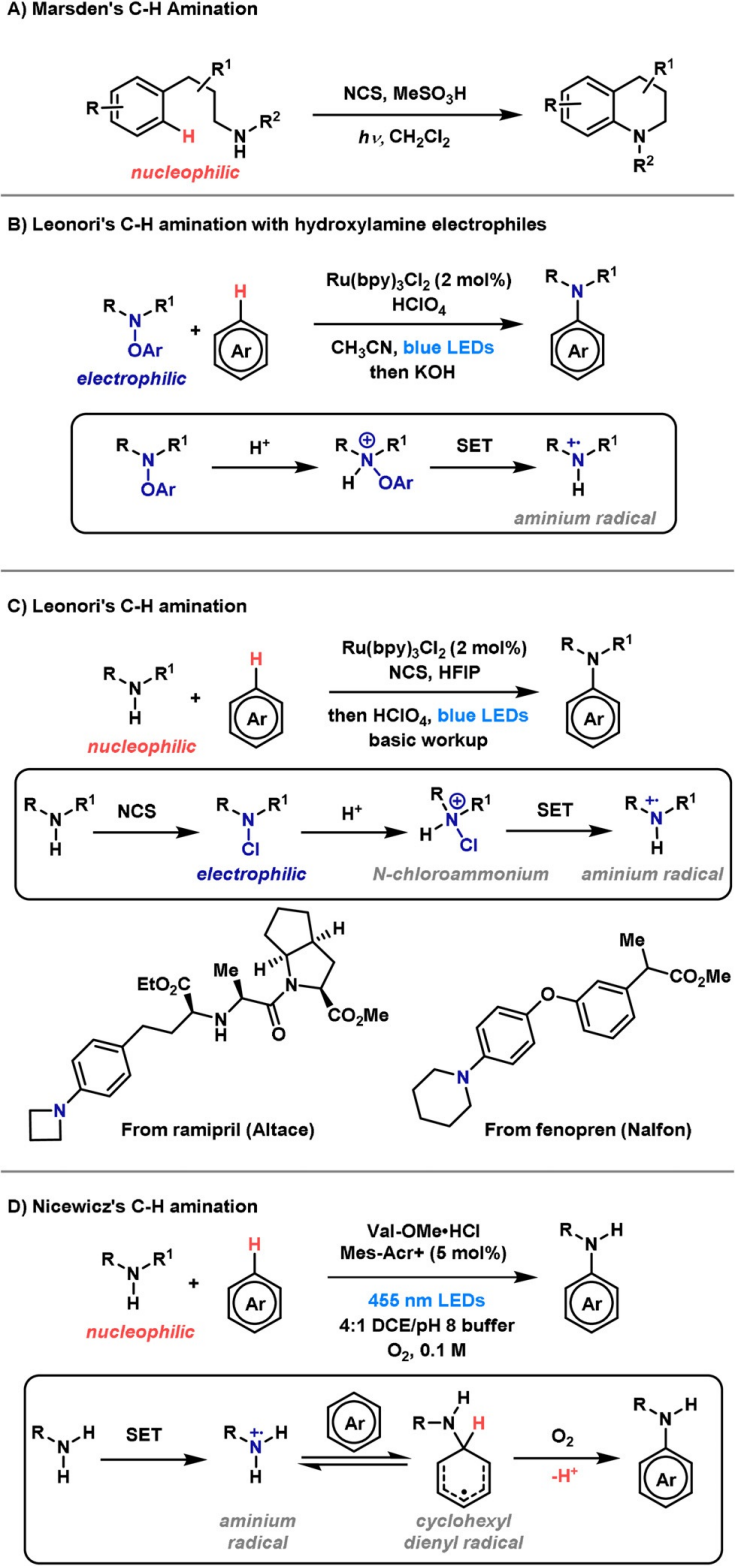
C‐H amination strategies with electrophilic radicals. Ar=aryl, HFIP=hexafluoro‐2‐propanol, Mes‐Acr+=9‐mesityl‐10‐methylacridinium, Ru(bpy)_3_Cl_2_=tris(bipyridine)ruthenium(II) chloride, SET=single‐electron transfer, Val‐OMe⋅HCl=*L*‐valine methyl ester hydrochloride.

Marsden and co‐workers have reported an efficient method for intramolecular C‐H amination using aminium radicals (Scheme 27 A).^[332]^ The key advance is that the N‐chloroamine precursor is formed in situ by treatment of secondary amines with NCS.^[333]^ This method constitutes one of the first examples of a one‐pot “metal‐free” aryl C‐H amination, although it does require acidic reaction conditions (to aid protonate of the initially formed aminyl radical) and high‐energy UV radiation. Under basic conditions, photoactivation of N‐chloroamines affords nucleophilic aminyl radicals that can effect C‐H amination of electron‐poor heteroarenes. A striking intramolecular example of this chemistry was used by Sarpong and co‐workers to assemble the pentacyclic skeleton of the indole alkaloid arboflorine.^[334]^

Complementary photocatalytic aryl C‐H amination methods have been developed, and some of these use electrophilic nitrogen sources. For example, Leonori has shown that N‐O reagents of type **109** and **139** can be converted to aminium radicals using blue LEDs and Ru(bpy)_3_Cl_2_ (Scheme 27 B).^[335]^ The method is relatively mild, although an acid additive (HClO_4_) is still required, and the N‐component requires prefunctionalization. The latter issue has been addressed via the development of a double amine activation strategy (Scheme 27 C).^[336]^ Here, NCS is used to generate an N‐chloramine in situ prior to addition of the photocatalyst and acid, which facilitates aminium radical formation. The method offers good scope and often excellent regioselectivities. The power of this strategy was demonstrated by the late‐stage (diversity‐oriented) functionalization of several complex molecules.

Another conceptually elegant approach was reported by Nicewicz.[^337^, ^338^] Here, the aminium radical is generated directly from a primary amine or nitrogen‐containing heterocycle (rather than from an electrophilic aminating agent) using acridinium photoredox catalysis coupled with aerobic oxidation (Scheme 27 D). Importantly, no acid is required for this transformation, enabling C‐H amination in the presence of acid‐sensitive functional groups. This strategy is reliant on oxidation of the primary amine to the aminium radical species by the acridinium photocatalyst. The addition of the arene initiates cyclohexadienyl radical formation, which is then rearomatized by molecular oxygen, thus generating the C‐H amination product. Although this method requires no prefunctionalization, at the current stage it is limited in scope with respect to the amine partner.

The direct installation of the NH_2_ functionality can also be achieved by C‐H amination processes involving electrophilic hydroxylamine‐based reagents.[^325^, ^339^, ^340^, ^341^] Here, metal‐catalyzed or metal‐mediated homolysis of the weak N−O bond generates the electrophilic aminium radical (^+.^NH_3_), which can engage the arene to deliver the aminated (NH_2_) product. This approach has found application in the synthesis of complex molecules; notably, Sanford's Ti^III^‐mediated arene C‐H amination was employed in the multigram preparation of a key intermediate in the synthesis of tamibarotene.^[339]^

The methodologies outlined in this section demonstrate the use of conventional and photocatalytic methods for the generation of aminium radicals, which are able to engage in C‐H amination processes, often removing the need for the prefunctionalization of starting materials. Although a useful method for the generation of aryl C−N bonds, the scope of these processes is limited in comparison to the classical Buchwald–Hartwig, Chan–Lam and Ullmann cross‐couplings; however, the development of new methods for the generation of aminium radicals has opened up this approach as a useful complementary strategy for the synthesis of aryl C−N bonds.

### 6.3 Metal‐Free Aziridinations

The aza‐MIRC aziridination process described previously (Section 3) is a simple and appealing aziridination process. However, it is not stereospecific and it is limited to electron‐poor α,β‐unsaturated systems. Most stereospecific aziridination processes require a transition metal and proceed via a nitrene intermediate.^[133]^ Metal‐free alternatives have been reported, but most require strong external oxidants and offer narrow scope.[^128^, ^342^, ^343^, ^344^, ^345^, ^346^, ^347^, ^348^] Mild and stereospecific metal‐free aziridinations are therefore of interest.

**Scheme 28:**
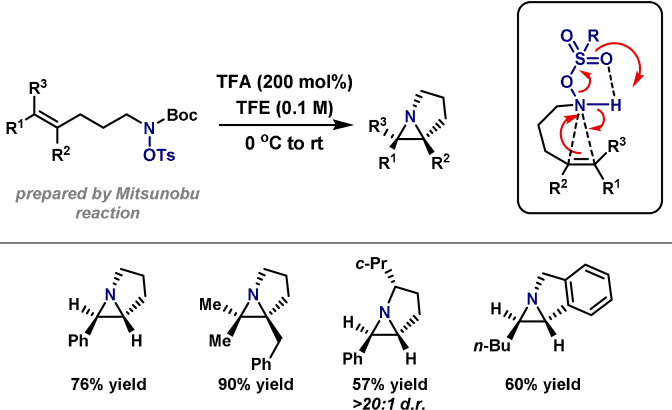
Bower's metal‐free, stereospecific aziridination. TFE= 2,2,2‐trifluoroethanol.

A recent report showed that aziridines can be accessed from non‐polarized alkenes by an aza‐Prilezhaev type process involving an electrophilic nitrogen source (Scheme 28).^[162]^ Here, the precursor is assembled by Mitsunobu reaction from the corresponding alcohol. Following the addition of acid to promote N‐Boc deprotection (TFA), an activated electrophilic hydroxylamine is able to engage alkenes in a process that appears to be an aza analogue of the *meta*‐chloroperoxybenzoic acid (*m*‐CPBA) epoxidation reaction, allowing for the stereospecific preparation of aziridine products. It was proposed that the transformation occurs via a butterfly‐like transition state, therefore the tosyl group is essential for successful reaction. Importantly, an external oxidant is not required, and this feature provides wide scope. The intriguing heterobicyclic aziridine products are primed for further diversification, and seem well suited to applications in total synthesis.

**Scheme 29:**
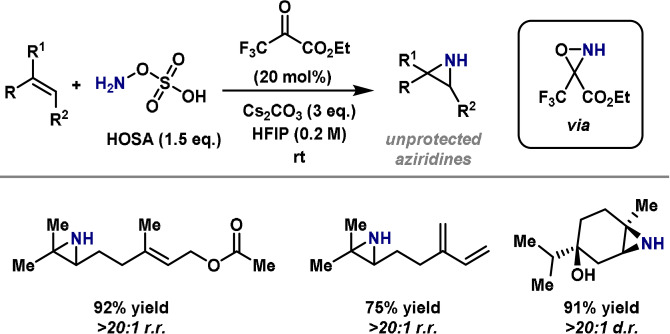
Kürti's organocatalytic aziridination. HOSA=hydroxylamine‐*O*‐sulfonic acid, r.r.=regiomeric ratio.

Falck, Kürti and co‐workers have developed a rhodium‐nitrene based method for the aziridination of alkenes. The protocol uses electrophilic hydroxylamine derivatives and, unusually, can provide NH aziridines.^[349]^ Similar conditions have been developed for aza‐Rubottom oxidation; interestingly, in many cases the Rh catalyst was not required, such that amination could be achieved under metal‐free conditions.^[350]^ More recently, a metal‐free alkene aziridination was reported, wherein unprotected aziridines were generated using an oxaziridine intermediate (Scheme 29).^[351]^ This reactive species was generated in situ from an electron‐poor ketone organocatalyst and a hydroxylamine‐based aminating agent (HOSA). The method allows the stereospecific aziridination of unactivated, nonconjugated olefins, and can be thought of as an aza analogue of the Shi epoxidation. Further, by using a chiral ketone organocatalyst (3‐trifluoroacetyl‐d‐camphor), promising levels of enantioinduction were achieved. The simplicity of this aziridination method bodes well for future applications in total synthesis.

## 7 Conclusion

This review has surveyed the landscape of electrophilic aminating agents in the context of total synthesis. The examples presented give an overview of available strategies, and also highlight the relative merits and disadvantages of each approach. The exact choice of reaction conditions and electrophilic aminating agent allows selection between diverse reaction manifolds. As such, a wide range of specific transformations can be conducted, and these often function as key assembly steps en route to complex targets. The umpoled reactivity of electrophilic aminating agents enables the reaction of typically unreactive bonds, as well as the formation of densely functionalized and sterically hindered tetrasubstituted stereocenters. Often these processes employ simple, achiral starting materials. As can be seen, electrophilic aminating agents offer a complementary strategy to conventional nucleophilic amination processes. In certain contexts, the umpoled approach is a superior option because it can minimize step count or enable a more powerful disconnection. For these reasons, it is likely that the use of electrophilic aminating agents will become more prevalent in total synthesis.

## Conflict of interest

The authors declare no conflict of interest.

## Biographical Information

*Lauren O'Neil graduated from the University of Nottingham in 2016 with an MSci in Chemistry, during which she worked at Sygnature Discovery for a year, before carrying out her final year project with Dr. James Dowden. She then moved to Bristol to join the Chemical Synthesis CDT and joined the Bower group in June 2017. Her PhD project is concerned with the total synthesis of the hasubanan alkaloids*.

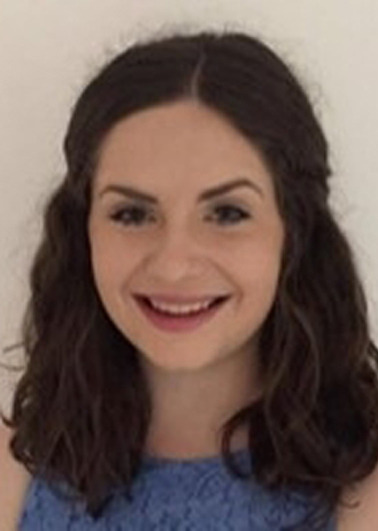

## Biographical Information

*John Bower obtained his MSci degree in Chemistry from the University of Bristol (2003), where he remained for his PhD studies (2007) with Prof. Timothy Gallagher. He had postdoctoral appointments with Prof. Michael Krische (University of Texas at Austin, 2007–2008) and Prof. Timothy Donohoe (University of Oxford, 2008–2010). In 2010, he commenced his independent career at the University of Bristol, where he was promoted to Professor in 2017. In 2020, he was appointed to the Regius Chair of Chemistry at the University of Liverpool*.